# Genetic structure of fragmented southern populations of African Cape buffalo (*Syncerus caffer caffer*)

**DOI:** 10.1186/s12862-014-0203-2

**Published:** 2014-11-01

**Authors:** Nathalie Smitz, Daniel Cornélis, Philippe Chardonnet, Alexandre Caron, Michel de Garine-Wichatitsky, Ferran Jori, Alice Mouton, Alice Latinne, Lise-Marie Pigneur, Mario Melletti, Kimberly L Kanapeckas, Jonathan Marescaux, Carlos Lopes Pereira, Johan Michaux

**Affiliations:** Departement of Life Sciences-Conservation Genetics, University of Liège, Liège, Belgium; Centre de Coopération Internationale en Recherche Agronomique pour le Développement (CIRAD), Montpellier, France; Fondation Internationale pour la Gestion de la Faune (IGF), Paris, France; Centre de Coopération Internationale en Recherche Agronomique pour le Développement (CIRAD)-RP-PCP, University of Zimbabwe, Harare, Zimbabwe; Mammal Research Institute, Department of Zoology and Entomology, University of Pretoria, Pretoria, South Africa; Department of Biological Sciences, University of Zimbabwe, Harare, Zimbabwe; Department of Animal Science and Production, Botswana College of Agriculture, Gaborone, Botswana; Institut des Sciences de l’Evolution-CNRS-IRD, Université de Montpellier 2, Montpellier, France; Department of Parasitology, Faculty of Veterinary Medicine, Kasetsart University, Bangkok, Thailand; Research Unit in Environmental and Evolutionary Biology, University of Namur, Namur, Belgium; Independent researcher, Via Di Villa Chigi, Rome, Italy; Department of Genetics and Biochemistry, Clemson University, Clemson, USA; Wildlife Conservation Society, New York, USA

**Keywords:** *Syncerus caffer caffer*, Population genetics, Genetic structure, Translocation, Southern Africa, Conservation implications

## Abstract

**Background:**

African wildlife experienced a reduction in population size and geographical distribution over the last millennium, particularly since the 19^th^ century as a result of human demographic expansion, wildlife overexploitation, habitat degradation and cattle-borne diseases. In many areas, ungulate populations are now largely confined within a network of loosely connected protected areas. These metapopulations face gene flow restriction and run the risk of genetic diversity erosion. In this context, we assessed the “genetic health” of free ranging southern African Cape buffalo populations (*S.c. caffer*) and investigated the origins of their current genetic structure. The analyses were based on 264 samples from 6 southern African countries that were genotyped for 14 autosomal and 3 Y-chromosomal microsatellites.

**Results:**

The analyses differentiated three significant genetic clusters, hereafter referred to as Northern (N), Central (C) and Southern (S) clusters. The results suggest that splitting of the N and C clusters occurred around 6000 to 8400 years ago. Both N and C clusters displayed high genetic diversity (mean allelic richness (*A*_*r*_) of 7.217, average genetic diversity over loci of 0.594, mean private alleles (*P*_*a*_) of 11), low differentiation, and an absence of an inbreeding depression signal (mean *F*_*IS*_ = 0.037). The third (S) cluster, a tiny population enclosed within a small isolated protected area, likely originated from a more recent isolation and experienced genetic drift (*F*_*IS*_ = 0.062, mean *A*_*r*_ = 6.160, *P*_*a*_ = 2). This study also highlighted the impact of translocations between clusters on the genetic structure of several African buffalo populations. Lower differentiation estimates were observed between C and N sampling localities that experienced translocation over the last century.

**Conclusions:**

We showed that the current genetic structure of southern African Cape buffalo populations results from both ancient and recent processes. The splitting time of N and C clusters suggests that the current pattern results from human-induced factors and/or from the aridification process that occurred during the Holocene period. The more recent S cluster genetic drift probably results of processes that occurred over the last centuries (habitat fragmentation, diseases). Management practices of African buffalo populations should consider the micro-evolutionary changes highlighted in the present study.

**Electronic supplementary material:**

The online version of this article (doi:10.1186/s12862-014-0203-2) contains supplementary material, which is available to authorized users.

## Background

In the context of recent global changes, the combined effects of human-induced factors (human population growth and subsequent habitat degradation/land conversion and cattle-borne diseases) and climatic fluctuations have had a marked impact on the population size and geographical distribution of African wildlife [1,2]. Consequently, African wildlife populations now tend to be confined within a network of protected areas, exposing them to the risk of natural gene flow disruption. Population fragmentation is a major challenge for long-term conservation because this process can induce genetic erosion, inbreeding depression and reduce the evolutionary potential of the species [3-8]. In this setting, genetics can provide key information to help identify conservation priorities and adequate management strategies. Genetic diversity, minimum population size and connectivity within meta-populations are the main indicators of the genetic health of a given population complex. Genetics can drive decision-making processes regarding conservation management (e.g. reintroduction, reinforcement and exchange of breeding individuals) to offset the negative effects of the above-mentioned population fragmentation process.

The southern African Cape buffalo (*Syncerus caffer caffer* - Sparrman 1779) was chosen as a model to study the impact of population fragmentation on the genetic diversity of large mammals in southern Africa. The African Cape buffalo is a key species in African savanna ecosystems due to its high contribution to the herbivore biomass. It is also a major attraction for the wildlife-viewing and hunting industries [9]. Like numerous other savanna species, the African buffalo has suffered major population losses over the last century. Historically widespread across sub-Saharan Africa, the species range has gradually been shrinking. Currently, around 75% of the global Cape buffalo population is considered to be located inside protected areas (PAs), with some populations completely isolated in tiny areas due to the presence of fences and/or nearby intensive human activities [10].

Besides currently suffering from habitat loss and consumptive uses, the African buffalo has also been considerably affected at the continental scale by the onset of the rinderpest epidemic in Africa at the end of the 19^th^ century [11]. Consequently, the drastic reduction in population size (i.e. bottleneck) associated with the fragmentation of a supposedly panmictic population could have been genetically marked by a decrease in allelic diversity, and later in heterozygosity [12]. However, despite high reported mortality rates at the continental scale, and according to the findings of numerous genetic studies, the rinderpest epidemic seems to have had little impact on the genetic diversity of the African Cape buffalo in terms of allelic or haplotype diversity [13-17]. The Cape buffalo was shown to display a large population size, considerable within-population genetic diversity, a high dispersal capacity and low population differentiation throughout eastern and southern Africa [9,13,15,18]. High genetic diversity paired with buffalo heterozygosity levels similar to those found in other species indicate that the demographic bottleneck due to rinderpest epidemics did not seem to result in a genetic bottleneck [13,15,16,19]. The resilience of African Cape buffalo populations to the rinderpest pandemic could be explained by the very large ancestral population size [13,20], relatively high intrinsic rate of increase of the species [21], good dispersal potential, and high degree of behavioural plasticity [22,23]. All available data indicate that the African Cape buffalo is likely a vagile species with one of the lowest levels of genetic differentiation among all large African mammals. This strongly suggests high gene flow between populations in the past [13,15,24-26].

In the early 20^th^ century, the natural connectivity between populations still enabled gene flows between recovering African Cape buffalo populations. This resulted in the re-establishment of genetic variability through the re-introduction of rare alleles, probably distorting the signal regarding the demographic bottleneck linked with the continental rinderpest epidemic [19]. However, throughout the 20^th^ century, habitat management (e.g. fencing) and sanitary measures (e.g. culling) adopted to control animal diseases tended to increase the fragmentation of buffalo populations [27,28]. Recent studies have suggested that the subsequent population size reduction and gene flow disruption are now serious enough to impact the genetic structure of buffalo populations [16]. The reduced level of gene flow leads to significant differentiation among populations, increased by the evolutionary processes of genetic drift and selection. Genetic drift was shown to have a more marked effect on buffalo populations in smaller areas [22]. In addition, these populations restricted to smaller areas seem also exposed to greater genetic erosion [22]. For example, correlations between protected areas and genetic variability indices have demonstrated that Cape buffalo populations in smaller parks displayed signs of genetic erosion in Kenya [22]. At this location, Cape buffalo populations that were able to move outside PAs in a low human density landscape displayed a weaker genetic structure in comparison to populations surrounded by high-density human communities. The susceptibility of a species to population fragmentation may thus be highly determined by its capacity to coexist with humans. Moreover, recent studies have shown that, with the increasing fragmentation of natural ecosystems in East Africa, the disruption of natural seasonal movements in response to seasonal variations in food availability and rainfall [26,29,30] has also impacted the genetic diversity of the African Cape buffalo [16].

Despite those findings, few studies have investigated the impact of African Cape buffalo population size reductions and gene flow disruption in southern African sub-regions and at a large geographical scale. Available data are mainly related to eastern African populations and/or a limited number of protected areas (PAs) [13-16,18,19,22,31]. To identify potential barriers to gene flow and management units, large-scale studies on the genetic structure between PAs are essential for the sustainable conservation of the species.

In this study, we assessed the “genetic health” of free ranging southern African Cape buffalo populations (*Syncerus caffer caffer*) and investigated the causal factors of their current genetic structure. We thus used 14 autosomal and 3 Y-chromosomal microsatellite markers to analyse 264 buffalo samples from 19 different locations in southern Africa.

## Methods

### Sampling and ethics statement

**Figure 1:**
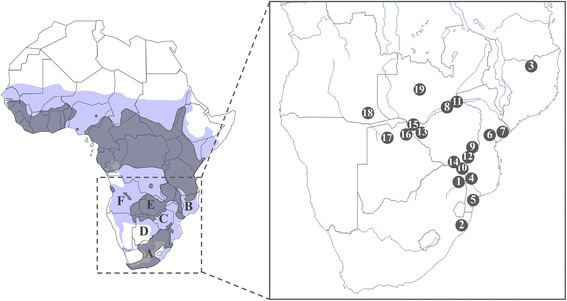
**Map of Africa representing the 19 sampling localities of** ***S. c. caffer*** **analysed in this study.** Grey shapes on the map represent the actual distribution of the African buffalo according to the IUCN Antelope Specialist Group, 2008. Blue shapes represent past distributions according to Furstenburg 1970–2008 (personal unpublished field notes). **A**. South Africa, **B**. Mozambique, **C**. Zimbabwe, **D**. Botswana, **E**. Zambia, **F**. Angola, 1. Kruger, 2. Hluhluwe-iMfolozi, 3. Niassa, 4. Limpopo, 5. Manguana, 6. Gorongosa, 7. Marromeu, 8. Nyakasanga, 9. Malilangwe, 10. Crooks Corner, 11. Mana Pools, 12. Gonarezhou, 13. Hwange, 14. Sengwe, 15. Victoria Falls, 16. Chobe, 17. Okavango Delta, 18. Angola, 19. Zambia.

Our collection of samples was compiled in collaboration with researchers having the required permits from the relevant national departments: the IGF foundation (Fondation Internationale pour la Gestion de la Faune- France) obtained authorization from the Department of Conservation of the Gorongosa National Park (GNP- Mozambique); CIRAD (Centre de Coopération Internationale en Recherche Agronomique pour le Développement – France, Botswana) obtained the relevant permits from the parks and wildlife management authorities in Bostwana, Mozambique, South Africa and Zimbabwe; Mario Melletti obtained the relevant permits to export samples from the wildlife management authorities of Zimbabwe and the University of Pretoria (South Africa) from the Hluhluwe-iMfolozi National Park. All partners obtained the ethical approval from their institution for the sampling procedure. The animal sampling protocols did not induce pain or distress according to the Animal Care Resource Guide (USDA category C). To facilitate the procedure, sampling of peripheral tissue (i.e. ear) or hair required the capture of buffalo through chemical immobilisation. The animals were released under veterinary supervision in favourable conditions. A total of 264 *S. caffer caffer* samples were collected at 19 localities in 6 countries (Figure 1, Table 1). Hair and tissue samples were stored in 96% ethanol. Genomic DNA was extracted from samples using the DNeasy Tissue Kit (QIAGEN Inc.) according to the manufacturer’s protocol.

**Table 1:** **Overview of the genetic parameters at each sampling locality**

**Country**	**Sampling locality**	**Fig 1 ID**	**Area (km** ^**2**^ **)**	***N***_***TOT***_	***N***	***N***_***a***_	***P***_***a***_	***A***_***r***_	***H***_***O***_ **(SD)**	***H***_***E***_ **(SD)**	***F***_***is***_	**N cluster affiliation%**	**C cluster affiliation%**	**S cluster affiliation%**
South Africa	Kruger	1	19,485	40 920 [32]	26	99	0	1.636	0.659 (0.204)	0.671 (0.203)	0.012	0	96.2	3.8
South Africa	Hluhluwe-iMfolozi	2	960	4 000 [21]	20	53	2	1.545	0.536 (0.250)	0.533 (0.226)	0.001	0	0	100
Mozambique	Niassa	3	42,300	6 200 [33]	20	100	2	1.650	0.663 (0.180)	0.680 (0.226)	−0.012	95	5	0
Mozambique	Limpopo	4	11,230	200 [34]	6	57	0	1.653	0.617 (0.246)	0.653 (0.239)	0.069	0	100	0
Mozambique	Manguana	5	-	-	4	51	0	1.634	0.649 (0.282)	0.634 (0.234)	−0.021	25	75	0
Mozambique	Gorongosa	6	4000	360 (C. Pereira, Pers. Comm. - 2010)	7	61	0	1.671	0.626 (0.291)	0.671 (0.207)	0.091	0	100	0
Mozambique	Marromeu	7	11,270	> 10 300 [35]	21	76	0	1.639	0.668 (0.228)	0.639 (0.225)	−0.062	95.2	4.8	0
Zimbabwe	Nyakasanga	8	1000	Unknown	2	36	1	1.806	0.542 (0.401)	0.597 (0.395)	0.063	100	0	0
Zimbabwe	Malilangwe	9	405	Unknown	20	97	2	1.659	0.636 (0.225)	0.659 (0.244)	0.008	35	55	10
Zimbabwe	Crooks corner	10	- Part of transfrontalier PA	-	13	81	1	1.634	0.581 (0.205)	0.634 (0.236)	0.058	0	100	0
Zimbabwe	Mana pools	11	6766	Unknown	10	90	1	1.700	0.641 (0.318)	0.700 (0.285)	0.120	60	40	0
Zimbabwe	Gonarezhou	12	7110	2 742 [36]	42	102	1	1.636	0.601 (0.256)	0.636 (0.255)	0.029	11.9	81	7.1
Zimbabwe	Hwange	13	14,651	Hwange and adjacent area: 24 500 [37]	6	63	0	1.598	0.557 (0.295)	0.628 (0.298)	0.052	33.3	66.7	0
Zimbabwe	Victoria falls	15	23		15	96	1	1.663	0.629 (0.307)	0.663 (0.261)	0.008	66.7	26.7	6.6
Zimbabwe	Sengwe	14	- transnational corridor between Kruger and Gonarezhou	-	8	73	0	1.646	0.571 (0.253)	0.646 (0.279)	0.076	0	75	25
Botswana	Chobe	16	11,700	Northern Botswana: 39 580 [38]	22	95	1	1.641	0.612 (0.250)	0.637 (0.262)	0.067	77.3	22.7	0
Botswana	Okavango delta	17	16,000		20	92	0	1.633	0.616 (0.254)	0.628 (0.257)	−0.003	90	5	5
Angola	Angola	18	No precise locality	-	1	22	3	1.583	0.571 (0.514)	0.571 (0.514)	-	-	-	-
Zambia	Zambia	19	No precise locality	-	1	24	0	1.667	0.714 (0.469)	0.714 (0.469)	-	-	-	-

This summarises the sample origin (country and sampling locality), size of the sampling locality in square kilometres, estimated number of buffaloes by aerial counts (*N*_*TOT*_), sample size per sampling locality involved in the present study (*N*) and mean values for number of alleles (*N*_*a*_), private alleles (*P*_*a*_), allelic richness (*A*_*r*_), observed (*H*_*O*_) and unbiased expected heterozygosity (*H*_*E*_) (and their standard deviations SD) and inbreeding coefficient (*F*_*is*_) across autosomal microsatellite loci. The affiliation of each sampling locality to each cluster, expressed in percentage, is also given in the last three columns (C; Central cluster, N; Northern cluster, S; Southern cluster).

### Microsatellite amplification and genotyping

The 264 samples used in this study were genotyped at 14 variable autosomal microsatellite loci (*TGLA227, TGLA263, ETH225, ABS010, BM1824, ETH010, SPS115, INRA006, BM4028, INRA128, CSSM19, AGLA293, ILSTS026, DIK020*- described by [39,40]). In addition, within this 264 samples set, all 86 males were also genotyped at three Y-chromosomal microsatellites (*UMN1113, INRA189, UMN0304*- described by [41]) (Additional file 1: Table S1). The three Y-chromosomal microsatellites were only used to reconstruct a minimum spanning network. Thirteen male samples failed to amplify at least at one Y-chromosomal microsatellite and were discarded for the minimum spanning network reconstruction (*N*_*Males*_ = 73). All those microsatellites were selected because they displayed good quality and high polymorphic amplification. The forward primer of each locus was 5’-end labeled with a fluorescent dye. Four multiplex sets were elaborated based on size limitations and amplification specificity: set 1 (*TGLA227, TGLA263, ETH225, ABS010, UMN0304*), set 2 (*BM1824, ETH010, SPS115, INRA189*), set 3 (*INRA006, BM4028, INRA128, CSSM19*), set 4 (*AGLA293, ILSTS026, UMN113, DIK020*). PCRs were carried out in 10 μl volumes containing between 0.1 and 0.2 μl of each 10 μM diluted primer (forward and reverse), 5 μl Multiplex PCR Master Mix (QIAGEN) and 2.5 μl DNA. Amplifications were performed in thermal VWR Unocycler using an activation step (94°C/15 min) followed by 30 cycles (denaturation at 94°C for 30 s, annealing at 57°C for 90 s, extension at 72°C for 60 s) and final extension step at 60°C for 30 min. PCR products were genotyped on a Applied Biosystems 3130XL Genetic Analyzer using 2 μl of amplified DNA, 12 μl of Hi-Di formamide and 0.4 μl of GeneScan-500 (LIZ) size standard (Applied Biosystem). Length variation determination was performed using GENEMAPPER 4.0 (Applied Biosystems).

### Microsatellite analysis

#### Genetic diversity and differentiation

MICRO-CHECKER 2.2.3 [42] was used to estimate the proportion of null alleles (NA) at each locus, calculated for each cluster (defined by STRUCTURE- see below), as well as the stutter errors or short allele dominance. The genotypes were then corrected relative to the results obtained with MICRO-CHECKER 2.2.3. Tests for linkage disequilibrium between loci for each sampling locality (SL) (Table 1), and the data fit to the Hardy-Weinberg equilibrium (HWE) proportions for each locus separately and over all loci for each SL, were performed with GENEPOP ([43] accessible online at: http://genepop.curtin.edu.au/). For both analyses, the Markov chain method was used with 1000 dememorizations, 1000 batches and 1000 iterations per batch. Fisher’s method for combining independent test results across SL and loci was used to determine the statistical significance of the test results.

Genetic diversity was assessed by calculating the expected (*H*_*E*_) and observed (*H*_*O*_) heterozygosities for each SL using both ARLEQUIN version 3.1 [44] and FSTAT 2.9.3 [45]. The *D*_*EST*_ estimator [46], as well as conventional pairwise *F*_*ST*_ statistics [47], were assessed using the online SMOGD application (http://www.ngcrawford.com/django/jost/) with 1000 bootstrap replicates [48], and ARLEQUIN 3.1, respectively. This allowed us to assess differences in allelic diversity among clusters. *D*_*EST*_ appeared to be more accurate than *G*_*ST*_ and its derivatives [46] for highly polymorphic markers such as microsatellites. *F*_*ST*_ was estimated for comparison with *D*_*EST*_, but also because it has been suggested to be more appropriate when both the sample size and number of applied loci are relatively low [49]. The hierarchical distribution of genetic variance among and within populations, based on *F*-statistics, was assessed using an analysis of molecular variance (AMOVA) performed with ARLEQUIN 3.1. The populations for the AMOVA analysis were defined according to the clustering results obtained with STRUCTURE 2.3. Allelic richness (*A*_*R*_ - [50]) was calculated for each SL using the rarefaction procedure implemented in FSTAT 2.9.3 [45], which also allowed estimation of the multi-locus *F*_*IS*_. The significance level was sequentially Bonferroni-adjusted for repeated tests [51].

A linear regression between patch areas (expressed as log km^2^) and different genetic indices of differentiation (mean pairwise *D*_*EST*_ and mean pairwise *F*_*ST*_) and diversity (allelic richness *A*_*R*_ and expected heterozygosity *H*_*E*_) for each SL were also performed to assess the effect of confinement within small enclosed protected areas (details for each SL are displayed in Table 1).

### Population structure

Bayesian clustering of microsatellite genotypes was performed using STRUCTURE 2.3, pooling individuals together independently of the spatial sampling, as described in the manual [52,53]. This software is implemented to cluster a sample set into a *K* number of groups so that each group is homogeneous. The number of clusters (*K*) was inferred by running 10 iterations for each *K* value from 1 to 20 using an admixture model with a burn-in of 1 × 10^5^ and MCMC repetition values of 1 × 10^6^. As the STRUCTURE software does not provide a statistical indication of the most likely *K*, results of the 10 iterations for each *K* value were summarized and averaged using CLUMPP 1.1.2 [54]. The *K* value that best fits the structure of the data set was revealed by the increasing likelihood of the data. It is chosen as the smallest value of *K* capturing the major structure in the data. The optimal number of clusters was then assessed based on correction as defined by Evanno [55].Visual output of the STRUCTURE 2.3. analysis was generated using DISTRUCT 1.1 [56]. Cluster membership of each sample was determined based on the average probability estimates provided by CLUMPP. The highest probability of each sample to belong to each cluster was used to determine its affiliation for the subsequent analyses. In the present study, the “cluster” term was attributed to define groups of individuals defined by STRUCTURE analysis, whereas “sampling locality” (SL) designates individuals sampled in the same protected area.

Moreover, three male-specific non-recombining microsatellites (*UMN0304, UMN1113* and *INRA189*) located on the Y-chromosome were selected for complementary analyses. Haplogroups were defined as a combination of the haplotypes defined for each of the three Y-chromosomal microsatellites, as described in the study of van Hooft *et al.* [41], while taking all three loci {n_1_, n_2_, n_3_} into account. Haplotypes for each microsatellite had to be defined because they could appear as multicopies on the Y-chromosome [41]. A minimum spanning network reconstruction was manually drawn by minimization of the number of mutations between haplotypes.

A factorial correspondence analysis (FCA), representing the proximity between each individual genotype in a 2D plot was performed based on the microsatellite allele frequencies using R software version 2.15.2 (R Development Core Team 2008) and the ade-4 package [57] using the 'fuzzygenet' function. Recent demographic bottlenecks were further investigated with BOTTLENECK 1.2 [58]. This software computes the average heterozygosity, which is compared to the observed heterozygosity to determine if a locus expresses a heterozygosity excess/deficit, according to the strict Stepwise Mutation Model (SMM [59]). Estimations were based on 1000 replications.

### Demographic history

For all subsequent analyses, software were run on two distinct microsatellite matrices: (i) a first one that included all individuals pooled together in each cluster determined by STRUCTURE 2.3, and (ii) a second matrix that only included individuals displaying a probability of belonging to each of the clusters over 0.9 (STRUCTURE 2.3). This measure was necessary to avoid bias associated with the high number of translocations that have taken place over the last decades in southern Africa (see Discussion).

The evolutionary history of *S. c. caffer* in southern Africa was inferred using coalescent-based DIYABC 1.0.4.45 beta software [60]. This program infers the population history by looking backwards in time to examine the genealogy of alleles until reaching the most recent common ancestor. The coalescent-based approximate Bayesian computation algorithm of DIYABC estimated the splitting time (in generation) as well as the effective population size of each tested cluster. Three clusters (or populations) were defined with STRUCTURE 2.3, with one almost exclusively composed of individuals from the Hluhluwe-iMfolozi PA. However, this last SL originated from an estimated population size of 75 individuals in 1929, after a population crash due to a rinderpest epidemic that eradicated around 95% of the South African buffalo population [20]. Only the two other clusters were considered in this analysis in order to avoid bias associated with a recent founder event and subsequent genetic drift.

**Figure 2:**
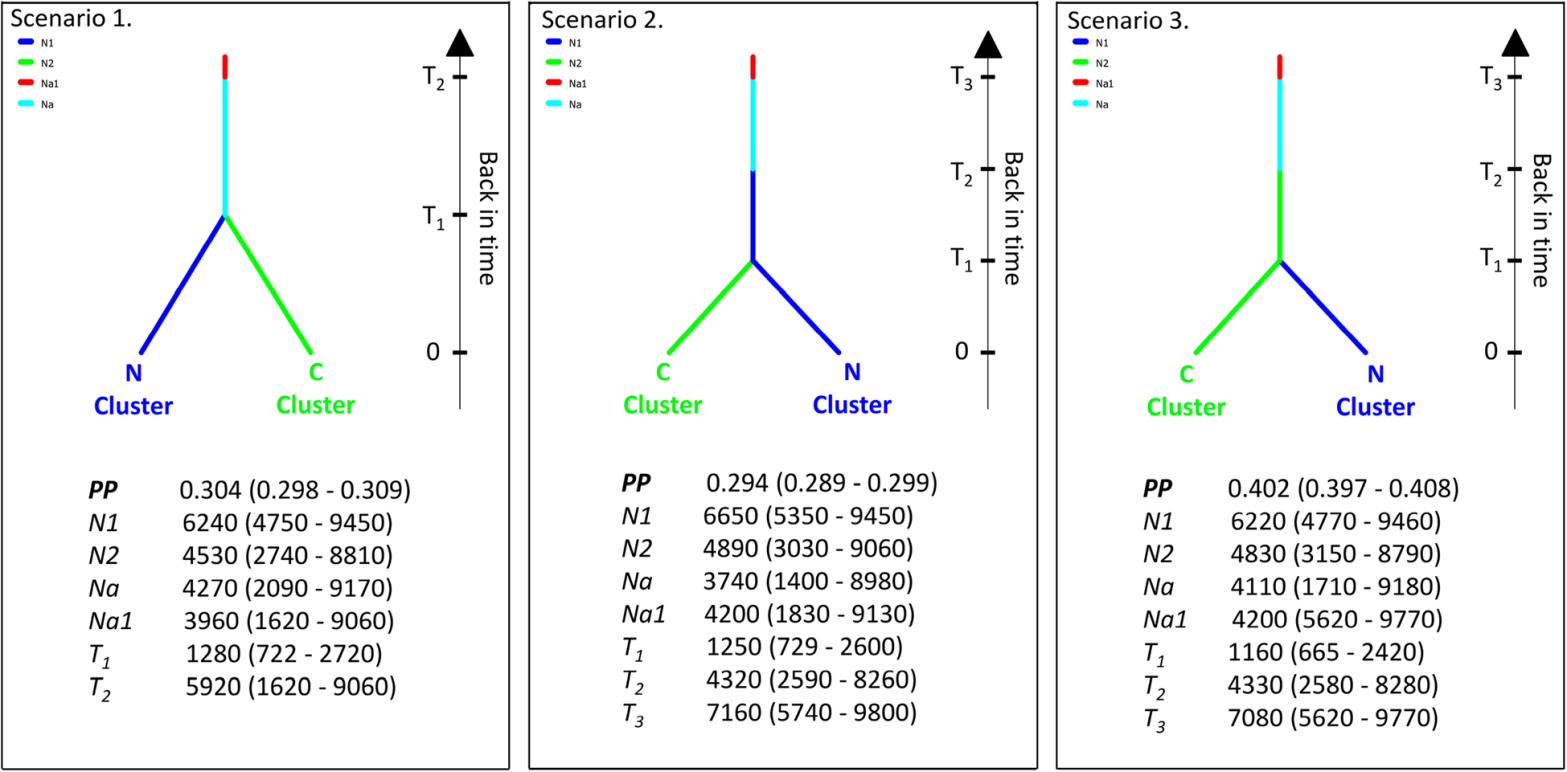
**Representation of three final competing scenarios tested with approximate Bayesian computation (ABC).** This analysis was based on a matrix including individuals displaying a probability of belonging to one of the two clusters over 0.9 (STRUCTURE software). N_*i*_ corresponds to effective population size of each cluster, and T_*i*_ corresponds to the time expressed in numbers of generations since divergence. The following conditions were considered: T_1_ < T_2_, T_2_ < T_3_, with 0 being the sampling date. Abbreviations are as follows: C; Central cluster, N; Northern cluster, PP; Posterior probability and their associated 95% confidence interval.

Alternative biogeographic divergence scenarios were inferred and compared using the DIYABC software package. In-depth information about scenario building procedure and alternative competitive scenario representations are available as additional file (Additional file 2: Figure S1A and S1B). Two runs were performed, a first one consisting of all scenarios (Additional file 2: Figure S1A and S1B), and a second one whereby only scenarios having the highest posterior probabilities were taken into consideration. The range and distribution of prior for parameters used to describe these scenarios (effective population size, time of splitting or merging events, and admixture rates) are given as additional file (Additional file 3: Table S2). For the second run, 3,000,000 datasets were simulated for each scenario (Figure 2) by building a reference table from a specified set of prior parameter distributions. A principal component analysis (PCA- DIYABC, Additional file 4: Figure S2) was performed on the first 30,000 simulated datasets to check if the set of scenarios and the prior distributions of their parameters were able to generate datasets similar to the observed dataset. A normalized Euclidean distance between each simulated dataset of the reference table and the observed dataset was calculated to identify the most likely scenario. To estimate the relative posterior probability of each scenario, 1% of the closest simulated datasets was used in a logistic regression. The most likely scenario was the one with the highest posterior probability.

Similarly, an isolation by distance (IBD) analysis among and within the three clusters defined with STRUCTURE 2.3 [52] was performed with GENEPOP 4.1.2 [61]. The *Dσ*^*2*^ estimates (i.e. product of population density and axial mean square parent-offspring distance as defined by [62]), were calculated according to *b* = 1/(4π *Dσ*^*2*^) to estimate the signal strength. The value obtained (*Dσ*^*2*^) was inversely correlated with the IBD strength. The geographic distance was calculated using the logarithm of the Euclidean distance on GPS coordinates. *â*_*r*_ statistics were used to represent the genetic distance between pairs of individuals [61]. We tested the significance of the correlation using a Mantel test with 30,000 permutations [63].

Furthermore, MIGRATE 3.4.4 [64-66] was used on the three populations. This allowed us to estimate different historical demographic events and genetic parameters, including the effective population size, the extent to which the clusters interacted (i.e. (im)migration rate (*M*)), and the confidence intervals. This analysis was developed by measuring similarities among our three clusters based on optimized *F*_*ST*_-based measures. This software employs a Metropolis-Hastings Markov chain Monte Carlo algorithm (MH) to search through genealogies, and a likelihood ratio test to obtain estimates of theta (Θ) and *M*. It assumes a constant Θ for each population but a variable Θ between them (pairwise migration rate estimates). MIGRATE 3.4.4 was used with default parameters, with the first five runs including *F*_*ST*_- based statistics of Θ and *M* involving 10 short chains with 10,000 sampled genealogies and three long chains with 100,000 sampled genealogies. A second analysis with five additional runs was performed with the parameter estimates of Θ and *M* from the previous run as starting values. Run convergence was checked by computing the MCMC autocorrelation, effective sample size and by visual comparison of the consistency of the results of each of the independent analyses. The headcount of immigrants per generation was calculated according to x*N*_*e*_m = M*Θ, with x being the inheritance scalar, set at 4 for diploid species, *N*_*e*_ the effective population size, and m the mutation rate per generation and per locus. The effective population size was assessed assuming a mean mutation rate within a range of 4.5*10^−5^ to 15*10^−5^ per generation.

## Results

### Population structure

**Figure 3:**
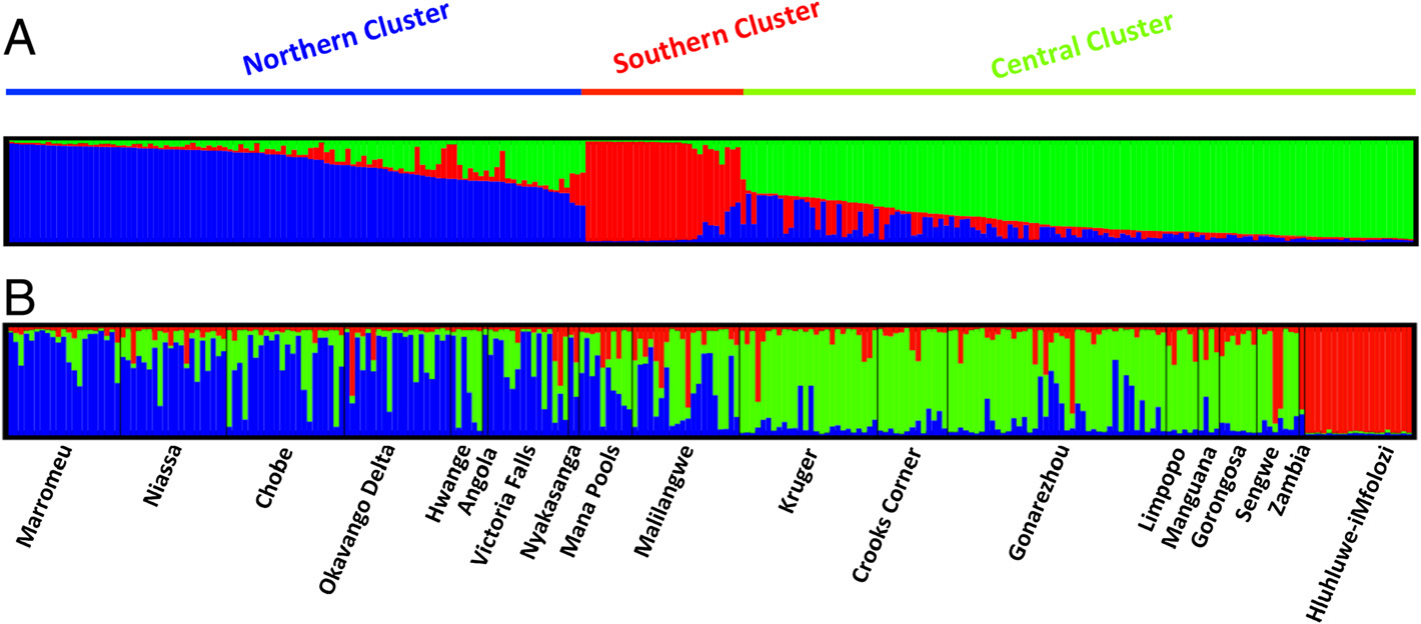
**Clusters inferred with STRUCTURE software, after the Evanno correction (** ***K*** **= 3).** The cluster membership of each sample is shown by the colour composition of the vertical lines, with the length of each colour being proportional to the estimated membership coefficient. The spatial representation is shown in Figure 4. **A**. Representation of the 3 clusters identified with STRUCTURE; **B**. Representation of the cluster membership of each sample within each sampling localities.

**Figure 4:**
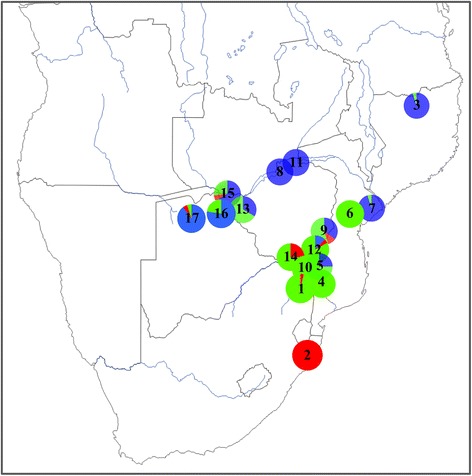
**Geographical representation of the three clusters assessed with STRUCTURE software (for SLs with** ***N***_***ind***_ **> 3).** Angola and Zambia are not represented due to the low sample sizes from those areas. Blue corresponds to the Northern cluster (N), green to the Central cluster (C) and red to the Southern cluster (S). The sampling localities are also given: 1. Kruger, 2. Hluhluwe-iMfolozi, 3. Niassa, 4. Limpopo, 5. Manguana, 6. Gorongosa, 7. Marromeu, 8. Nyakasanga, 9. Malilangwe, 10. Crooks Corner, 11. Mana Pools, 12. Gonarezhou, 13. Hwange, 14. Sengwe, 15. Victoria Falls, 16. Chobe, 17. Okavango Delta.

The STRUCTURE 2.3 software output was interpreted using the *ΔK* method, as described by Evanno [55]. The highest *ΔK* was for *K* = 3 (Figure 3 and Additional file 5: Figure S3), suggesting the existence of three clusters in our dataset (*ΔK* = 262.2). These clusters were considered as different “populations” in the subsequent analyses. The proportion of each cluster within every sampled locality is represented in Figure 4. The first cluster –N- (in blue in Figure 4) mainly appeared in samples collected in the northern section of the study area (all samples of Nyakasanga and Mana Pools, a large part of the samples from Niassa, Marromeu, Victoria Falls, Okavango Delta and Chobe, as well as from Hwange, to a lesser extent). The second cluster –C- (in green on Figure 4) appears in the central section of the study area, and is represented in very high proportions in the sets of samples from Kruger, Sengwe, Manguana, Limpopo and Hwange. The third cluster –S- (in red on Figure 4) essentially includes samples from Hluhluwe-iMfolozi, although residual shared loci were observed at other sampling localities.

Note that most clusters appeared to be admixed (Figure 4). This could partially be due to translocation operations since quite a large number of buffaloes have been captured and translocated in recent years. For example, the original buffalo population of Gorongosa (number 6 on Figure 4) was nearly extinct and recently reinforced with 186 buffaloes translocated from Kruger and adjacent Limpopo PAs (2006–2011; C. Lopes Pereira, pers. com.). In the present study, all samples from Gorongosa were reintroduced buffaloes from those latter two localities (C. Lopes Pereira, pers. comm.).

**Figure 5:**
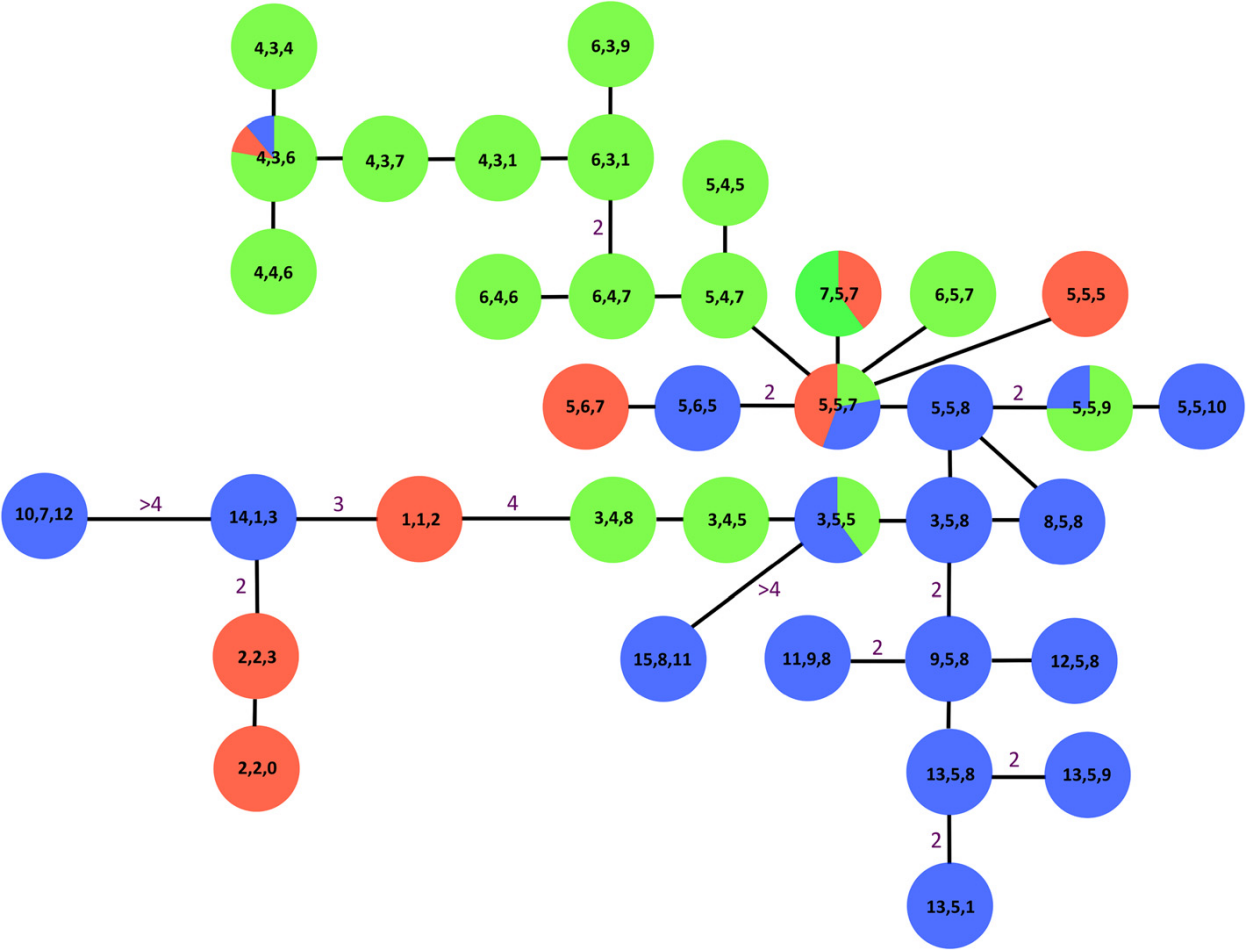
**Minimum spanning network reconstruction based only on the three Y chromosomal microsatellites.** It was manually drawn by minimization of the number of mutations between haplotypes. The three numbers in boxes refer to the different haplotypes at microsatellites *UMN0304, UMN113*, and *INRA189,* respectively, as described in van Hooft *et al.* [41]. Our dataset was standardized and pooled with the data of van Hooft *et al.* [41] that were absent from our sampling, i.e. (4, 3, 4), (4, 3, 7), (4, 3, 1), (6, 3, 1), (6, 3, 9), (5, 4, 5), (5, 4, 7), (5, 6, 7), (5, 5, 5), (2, 2, 0) and (3, 4, 8) from the Kruger and Hluhluwe-iMfolozi PAs. Numbers on the branches indicate the minimum number of mutations (when absent, only one mutation was observed between haplogroups). Box colours refer to the three clusters identified with the autosomal microsatellites using STRUCTURE software. The three most frequent haplogroups were (2, 2, 3), (5, 5, 7) and (4, 3, 6) (see Table 2).

A similar general genetic pattern can be noted on the Y-chromosomal microsatellite minimum spanning network reconstruction (Figure 5). Seventy-three male specimens were genotyped with the Y chromosomal microsatellites. Twenty-six haplogroups were observed in southern Africa, pooled with the haplogroups identified by van Hooft *et al.* [41], with each haplogroup being a unique combination of the haplotypes defined for each of the three Y-chromosomal microsatellites, i.e. {n _UMN1113 haplotype_, n _UMN0304 haplotype_, n _INRA189 haplotype_} (Table 2). More precisely, we respectively observed 15, 9, and 12 haplotypes at the *UMN1113*, *UMN0304* and *INRA189* loci. The detailed polymorphic loci of each of these microsatellites as well as their frequencies are reported in Table 2. Haplogroups {5, 5, 7} and {2, 2, 3} were present at the highest frequencies, namely 0.123 and 0.110, respectively, followed by {4, 4, 6} with 0.082, exclusively from Sengwe, and {3, 5, 5} with 0.069 found in Chobe, Nyakasanga, Malilangwe and Mana Pools. The other haplogroups did not overstep a frequency of 0.055. Moreover, only haplogroups {5, 5, 7} and {4, 3, 6} were observed in the three clusters. Figure 5 shows moderate structuring of the two main clusters (i.e. the Northern (blue) and the Central (green) clusters, identified by STRUCTURE 2.3). This was not the case for the more disparate Southern cluster (red).

**Table 2:** **Haplotype designation, haplogroup determination and their frequencies for the three Y-chromosomal microsatellite loci (** ***UMN0304, UMN1113*** **and** ***INRA189*** **, N**_**Males**_ **= 73)**

***UMN0304*** **alleles combination**	***UMN0304*** **haplotype designation**	***UMN113*** **allele combination**	***UMN113*** **haplotype designation**	***INRA189*** **allele combination**	***INRA189*** **haplotype designation**	**Haplo-group**	**Freq**
215-225	1	131	1	148-153-158-166	2	{1,1,2}	0,014
217-227	2	133	2	148-153-158-164	3	{2,2,3}	0,110
205-215-225	3	131-157	4	148-162	5	{3,4,5}	0,014
205-215-225	3	131-155	5	148-158	5	{3,5,5}	0,069
205-215-225	3	131-155	5	148-158	8	{3,5,8}	0,027
205-217	4	131-159	3	160	6	{4,3,6}	0,123
205-217	4	131-157	4	160	6	{4,4,6}	0,082
205-215-223	5	131-155	5	148-160	7	{5,5,7}	0,123
205-215-223	5	131-155	5	148-158	8	{5,5,8}	0,014
205-215-223	5	131-155	5	151-156	9	{5,5,9}	0,055
205-215-223	5	131-147-155	6	148-158	5	{5,6,5}	0,027
205-217-223	6	131-157	4	160	6	{6,4,6}	0,014
205-217-223	6	131-157	4	148-160	7	{6,4,7}	0,027
205-217-223	6	131-155	5	148-160	7	{6,5,7}	0,014
215-223	7	131-155	5	148-160	7	{7,5,7}	0,041
205-215	8	131-155	5	148-158	8	{8,5,8}	0,014
205-217-227	9	131-155	5	148-158	8	{9,5,8}	0,014
217-223	10	131-133-147-155	7	137-148-158-162	12	{10,7,12}	0,014
205-215-227	11	133-155	9	148-158	8	{11,9,8}	0,055
205-221-227	12	131-155	5	148-158	8	{12,5,8}	0,027
205-213-227	13	131-155	5	151-156	9	{13,5,9}	0,014
205-213-227	13	131-155	5	151-160	1	{13,5,1}	0,014
205-213-227	13	131-155	5	148-158	8	{13,5,8}	0,014
217-225-227	14	131	1	148-153-158-164	3	{14,1,3}	0,027
213-225	15	133-147-155	8	151-153-162	11	{15,8,11}	0,014
205-215-223	5	131-155	5	148-151	10	{5,5,10}	0,041

Each haplotype was attributed a number to designate the combination of alleles for each of the three loci because they can appear as multicopies on the Y-chromosome. The haplogroup is thus defined as the combination of the haplotypes, written as {n _UMN1113 haplotype_, n _UMN0304 haplotype_, n _INRA189 haplotype_}, where n _UMN1113 haplotype_ = 1,…,15, n _UMN0304 haplotype_ = 1,…,9, and n _INRA189 haplotype_ = 1,…,12.

**Figure 6:**
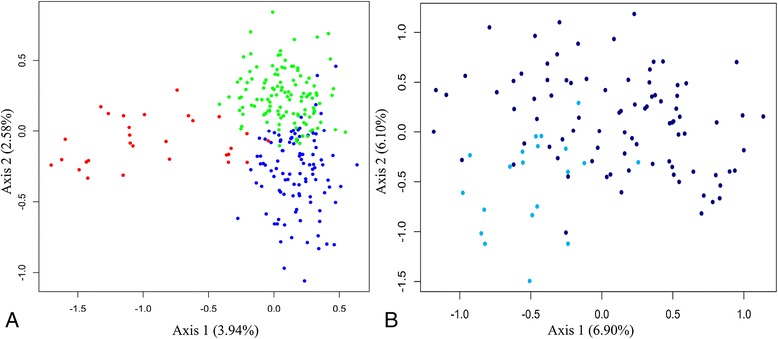
**Plot of the factorial correspondence analysis (FCA). A**.: Global FCA including the three clusters. Red: Southern cluster, Green: Central Cluster, Blue: Northern Cluster. **B**.: FCA on the Northern cluster. Turquoise: Marromeu samples, Dark blue: other populations included in the Northern cluster.

Finally, the three clusters identified by STRUCTURE 2.3 appeared to be relatively well defined, with the Southern cluster being more clearly separated from the two others based on visual assessment of the FCA plot (Figure 6A.). Furthermore, within the Northern cluster, a very smooth separation appeared between the Marromeu SL compared to all other SLs included in this cluster (Figure 6B).

### Genetic diversity

As highlighted in previous studies using the same microsatellites in different geographical areas [39-41], all microsatellites were detected as being polymorphic in each of the SL of the southern African sub-region. The number of alleles per microsatellite ranged from 2 to a maximum of 23 (Additional file 1: Table S1, Additional file 6: Table S3). The mean number of alleles across loci ranged from 6.3 in the Southern cluster to 9.5 in the Northern cluster. A significant presence of null alleles was detected for microsatellites *BM1824* and *INRA006* in the Northern cluster, and for *TGLA227, BM1824* and *AGLA293* in the Central cluster. They were corrected as suggested in the MICRO-CHECKER 2.2.3 user manual. A Hardy-Weinberg exact test performed on each SL at each loci showed no deviation from the expected frequencies after Bonferroni’s correction. One pair of loci (*SPS115* and *DIK020*) was found to show significant linkage disequilibrium in two populations (Northern and Central clusters). No single microsatellite marker exhibited an especially high number of private alleles (Additional file 1: Table S1).

Overall, the genetic variation level was high in all clusters. Inbreeding coefficients (*F*_*IS*_) were low to moderate but significant for all three clusters (Table 3). This index showed more variation when calculated for each SL separately, with a high *F*_*IS*_ value (0.12) for Mana Pools (Table 1). The microsatellite genetic diversity analysis showed that pairwise differences and *F*_*ST*_ were significant between all three clusters (Table 4), which was not always the case between animals from different SLs within each of the clusters (Table 5). Similar pattern were observed regarding pairwise *F*_*ST*_ and *D*_*EST*_ values among SLs and clusters: lower genetic differentiation among the Central SLs and low/moderate differentiation among the Northern SLs (Table 5). Moreover, we found higher genetic differentiation between the Northern/Central clusters and the Southern cluster (*F*_*ST*_ and *D*_*EST*_ -Tables 4 and 6). The mean observed heterozygosity ranged from 0.56 to 0.66, and was within the range of the main expected heterozygosity reported in Table 3. Allelic richness reached up to 7.8 in the Northern cluster (Table 3). The average number of alleles, average genetic diversity over loci, allelic richness, and heterozygosity were similar in the Northern and Central clusters. Interestingly, the results of the Wilcoxon test under a stepwise mutation model performed using the BOTTLENECK 1.2 software package indicated that the Southern and Central clusters had undergone a recent bottleneck (probability of 0.007 and 0.032, respectively), whereas the Northern cluster did not (probability of 0.191).

**Table 3:** **Overview of the genetic parameters at each cluster**

**Cluster**	***N***	***N***_***Males***_	***N***_***Females***_	***N***_***a***_ ***(SD)***	***P***_***a***_	***A***_***r***_	***H***_***O***_ **(SD)**	***H***_***E***_ **(SD)**	***F***_***IS***_	**Average genetic diversity over loci (SD)**
Northern	108	35	73	9.500 (5.360)	13	7.834	0.656 (0.242)	0.668 (0.264)	0.016	0.608 (0.319)
Central	125	35	90	9.143 (5.614)	8	7.657	0.635 (0.213)	0.657 (0.224)	0.033	0.601 (0.318)
Southern	31	16	15	6.286 (3.518)	2	6.160	0.556 (0.208)	0.591 (0.205)	0.062	0.573 (0.303)

*N*: Number of samples, *N*_*a*_: Mean number of alleles, *P*_*a*_: Private alleles, *A*_*r*_: Allelic richness, *H*_*O*_: Observed heterozygosity, *H*_*E*_: Expected heterozygosity, *F*_*IS*_: Inbreeding coefficient, SD: Standard deviation.

**Table 4:** **Differentiation results obtained with ARLEQUIN software (calculated on the matrix including individuals displaying a probability of belonging to one of the three clusters over 0.9 (STRUCTURE software))**

	**Northern**	**Southern**	**Central**
Northern	6.841	1.330^***^	0.545^***^
Southern	0.172^***^	5.499	1.338^***^
Central	0.075^***^	0.175^***^	6.643

Below diagonal, *F*_*ST*_ value- Diagonal elements, average number of pairwise differences within population (PiX)- Above diagonal, corrected average pairwise difference (PiXY-(PiX + PiY)/2). ^***^indicates *P* < 0.0005.

**Table 5:** ***F***_***ST***_ **(Below diagonal- ARLEQUIN software) and** ***D***_***EST***_ **(Above diagonal- SMOGD software) for the 16 geographical populations**

	**1 (N)**	**2 (N)**	**3 (C)**	**4 (S)**	**5 (C/N)**	**6 (C)**	**7 (N)**	**8 (C)**	**9 (C)**	**10 (N)**	**11 (C)**	**12 (C)**	**13 (C)**	**14 (C)**	**15 (N)**	**16 (N)**
1 (N)	/	0.056	0.079	**0.247**	0.043	0.066	0.093	0.084	0.063	0.001	0.084	0.091	0.018	0.051	0.057	0.074
2 (N)	0.033 ^***^	/	0.058	**0.225**	0.001	0.076	0.001	0.007	0.075	0.019	0.065	0.078	0.009	0.086	0.090	0.001
3 (C)	0.041 ^***^	0.036 ^***^	/	**0.213**	0.001	<−0.0001	0.037	0.004	−0.010	0.007	0.015	<0.0001	<−0.0001	0.0002	0.105	0.060
4 (S)	**0.163** ^***^	**0.167** ^***^	**0.141** ^***^	**/**	**0.236**	**0.235**	**0.245**	**0.177**	**0.234**	**0.293**	**0.244**	**0.141**	**0.142**	**0.148**	**0.290**	**0.242**
5 (C/N)	0.037 ^*^	−0.007 ^ns^	0.026 ^*^	**0.196** ^***^	/	0.002	−0.0001	0.002	0.007	0.0001	0.016	0.024	<0.0001	0.037	0.064	0.009
6 (C)	0.042 ^*^	0.056 ^***^	0.010 ^ns^	**0.181** ^***^	0.056 ^***^	/	0.012	0.006	0.003	0.023	0.019	<0.0001	<0.0001	0.011	0.087	0.034
7 (N)	0.045 ^***^	0.006 ^ns^	0.033 ^***^	**0.157** ^***^	0.004 ^ns^	0.062 ^***^	/	0.019	0.050	0.006	0.041	0.081	0.008	0.066	0.083	0.005
8 (C)	0.044 ^***^	0.020 ^***^	0.014 ^*^	**0.130** ^***^	0.016 ^ns^	0.034 ^*^	0.022 ^*^	/	0.012	0.018	0.017	0.008	<−0.0001	0.021	0.118	0.012
9 (C)	0.049 ^***^	0.051 ^***^	−0.008 ^ns^	**0.175** ^***^	0.056 ^***^	0.024 ^ns^	0.059 ^***^	0.025 ^*^	/	0.003	0.005	−0.007	<−0.0001	0.001	0.116	0.062
10 (N)	0.016 ^*^	0.029 ^***^	0.021 ^*^	**0.163** ^***^	0.010 ^ns^	0.032 ^*^	0.021 ^ns^	0.027 ^*^	0.035 ^*^	/	0.021	0.002	0.010	0.013	0.081	0.009
11 (C)	0.037 ^***^	0.030 ^***^	0.008 ^*^	**0.154** ^***^	0.031 ^*^	0.013 ^ns^	0.031 ^***^	0.012 ^*^	0.013 ^ns^	0.025 ^*^	/	0.005	−0.008	0.005	0.111	0.047
12 (C)	0.046 ^*^	0.060 ^***^	0.002 ^ns^	**0.128** ^***^	0.053 ^ns^	0.005 ^ns^	0.060 ^*^	0.023 ^ns^	0.002 ^ns^	0.016 ^ns^	0.026 ^*^	/	0.003	0.020	0.112	0.046
13 (C)	0.037 ^*^	0.030 ^ns^	−0.015 ^ns^	**0.139** ^***^	0.029 ^ns^	0.007 ^ns^	0.029 ^ns^	−0.005 ^ns^	−0.009 ^ns^	0.029 ^ns^	−0.013 ^ns^	0.011 ^ns^	/	−0.0002	0.048	0.025
14 (C)	0.020 ^*^	0.036 ^***^	−0.010 ^ns^	**0.133** ^***^	0.043 ^ns^	0.030 ^ns^	0.043 ^*^	0.018 ^*^	−0.010 ^ns^	0.030 ^ns^	0.010 ^ns^	0.026 ^ns^	−0.012 ^ns^	/	0.109	0.065
15 (N)	0.034 ^***^	0.059 ^***^	0.064 ^***^	**0.195** ^***^	0.055 ^***^	0.067 ^***^	0.055 ^***^	0.064 ^***^	0.081 ^***^	0.050 ^***^	0.063 ^***^	0.073 ^***^	0.051 ^*^	0.064 ^***^	/	0.082
16 (N)	0.032 ^***^	−0.001 ^ns^	0.045 ^***^	**0.166** ^***^	−0.003 ^ns^	0.059 ^***^	0.008 ^ns^	0.012 ^*^	0.068 ^***^	0.022 ^***^	0.036 ^***^	0.060 ^***^	0.037 ^*^	0.047 ^***^	0.058 ^***^	/

These estimators were only computed where more than 5 samples were available. 1: Niassa, 2: Chobe, 3: Kruger, 4: Hluhluwe-iMfolozi (bold), 5: Hwange, 6: Sengwe, 7: Victoria Falls, 8: Malilangwe, 9: Crooks Corner, 10: Mana Pools, 11: Gonarezhou, 12: Limpopo, 13: Manguana, 14: Gorongosa, 15: Marromeu, 16: Okanvango Delta. Cluster affiliation of each sampling locality is also indicated in the table as follow: (N) for the Northern cluster, (C) for the Central cluster and (S) for the Southern cluster. Here, one SL is considered to belong to one cluster if there are more than 50% of SL’s individuals that belongs to this cluster. ^*^indicates *P* < 0.05, ^**^indicates *P* < 0.005, ^***^indicates *P* < 0.0005; ^ns^indicates non-significant.

**Table 6:** **Harmonic mean of** ***D***_***EST***_ **across loci between each of the three clusters (SMOGD software)**

	**Northern**	**Southern**	**Central**
Northern		0.281	0.137
Southern			0.276
Central			

This computation was performed on the matrix, including individuals displaying a probability of belonging to one of the three clusters over 0.9 (STRUCTURE software).

### Demographic history

The DIYABC 1.0.4.45 beta software package was used to determine which historical demographic scenario could best explain the observed microsatellite polymorphism. The approximate Bayesian computation approach was used on 16 distinct demographic scenarios (Additional file 2: Figure S1A and S1B), followed by a second analysis with only three demographic scenarios that presented the highest posterior probabilities (PP) in the first run [67,68]. The last three competing scenarios included in the second run revealed a similar general evolutive demographic pattern (Figure 2), namely a binary split without admixture events. After polychotomous logistic regression on the 1% closest simulated datasets to the observed one, the most likely scenario according to DIYABC 1.0.4.45 beta was scenario 3, as represented in Figure 2, with a PP of 0.402 and a confidence interval (95 CI) of 0.397–0.408. The PP of scenario 3 did not overlap with the two alternative scenarios. When comparing the posterior distribution of parameters of those three competitive scenarios, the differentiation time at time T_1_ and the effective population size estimates (N_1_ and N_2_) were all within same order of magnitude. The median (95% CI) of the estimated time since divergence (T_1_) between the N and C clusters was evaluated at about 1200 generations earlier (Figure 2). Assuming a generation time of 5–7 years, divergence time corresponds to the Holocene epoch (T_1_: 6000 to 8400 years ago). The effective population size estimates for the C and N clusters reached a mean of 4700 and 6400 individuals, respectively (Figure 2). The effective population sizes assessed with MIGRATE 3.4.4, assuming a mean mutation rate of 4.5*10^−5^ to 15*10^−5^ per generation, was estimated between 7000 to 25,000 for the Northern cluster, 600 to 2000 for the Southern cluster and 3000 to 10,000 for the Central cluster. In agreement with the summary statistics of DIYABC 1.0.4.45 beta and the previously estimated summary statistics described hereafter, there were no obvious differences between estimates of heterozygosity, genetic diversity and *F*_*ST*_.

Migration rate could not be assessed with the IM software (for more information- see Additional file 7: IM), probably linked to the very high variation percentage within clusters (87.40%) as compared to the variation among them (12.60%) (AMOVA). MIGRATE 3.4.4 was used for this purpose, immigration rate per generation being calculated according to N_e_m = (*M*_i →j_*Θ_j_)/4, with Θ_S_ = 0.38, Θ_N_ = 4.40, Θ_C_ = 1.81 and *M*_C→S_ = 4.17, *M*_S→N_ = 0.84, *M*_C→N_ = 2.56, *M*_S→C_ = 1.06, *M*_N→C_ = 5.01, *M*_N→S_ = 1.67. The effective number of immigrants per generation between clusters was low, from less than one migrant per generation (each 5–7 years) coming from the Southern towards the Central/Northern clusters, and vice versa (Nm_N→S_ = 0.16, Nm_S→C_ = 0.48, Nm_C→S_ = 0.40, Nm_S→N_ = 0.92), to approximately two migrants between the Central and Northern clusters (Nm_N→C_ = 2.27 and Nm_C→N_ = 2.81). The results between the two runs on the separate microsatellite matrices were similar.

Finally, an IBD analysis was performed on the two microsatellite matrices to test whether the geographic distance could explain the low number of migrants. Both runs indicated an absence of isolation by distance among clusters but a significant signal within the Northern and Central clusters (*Dσ*^*2*^_Central cluster_ = 9.95, *Dσ*^*2*^_Northern cluster_ = 2.69). The analysis was not performed on the third cluster because all samples in this cluster originated from a single protected area, i.e. Hluhluwe-iMfolozi. The signal in the Central cluster was not strong, as indicated by the relatively high *Dσ*^*2*^ value and by the slight slope of the regression of pairwise genetic statistics against the log distance. The slight regression slope indicated that the genetic distance between pairs of individuals was weakly correlated with the geographic distance between them. The signal was stronger in the Northern cluster. The absence of significant signal between the three clusters showed that the cluster generation was not driven by the geographic distance. Concerning the linear regression performed between the log habitat area and the different genetic differentiation indices and diversities for each SL, the *R*^*2*^ (coefficients of determination) were close to 0. This indicated an absence of any linear relationship between the log habitat areas and the different genetic indices (*A*_*R*_: *R*^*2*^ = 0.03, Mean *H*_*E*_: *R*^*2*^ = 0.05, Mean pairwise *D*_*EST*_: *R*^*2*^ = 0.04, Mean pairwise *F*_*ST*_: *R*^*2*^ = 0.05). Graphic representations are available as supplementary information (Additional file 8: Figure S4).

## Discussion

The present study provides new insight into the current genetic structure of buffalo populations in southern Africa, indicating the existence of three genetically and geographically distinct populations, or so-called meta-populations (Figure 4). The sampling covered a large part of the current distribution area of the southern African buffalo, thus ensuring robust analytical findings. We demonstrated that the three-cluster structuring did not result from isolation by geographical distance (IBD), but probably from other human and environmental factors. We further discuss the impact of translocations on the genetic structure of southern African buffalo populations, as well as the observed genetic diversities within and between each of the clusters/sampling locality (SL), from a wildlife conservation management standpoint.

### Demographic history of the Northern (N) and the Central (C) clusters

The time of splitting of the N and the C clusters was dated at about 6000 to 8400 years ago. This splitting time may be underestimated, given that the DIYABC algorithm assumes no migration between the scenario events [60]. The differentiation time may thus be an underestimation of the real splitting time. Nevertheless, our results are well corroborated by the findings of a previous study conducted by Heller *et al.* [31]. These authors compared Bayesian skyline plots of African buffalo samples from three localities (Zimbabwe/Bostwana, Ethopia and Kenya). A moderate and then accelerating buffalo population decline was highlighted over the course of the Holocene [31]. This decline suggests a major ecological transition between the Palaeolithic, during which the buffalo population expanded, and the Neolithic, during which the buffalo population declined [31]. This was probably induced by two concomitant causal factors, i.e. climatic changes and explosive human population growth [31].

In the first case, climatic changes were proposed to have strongly impacted buffalo population dynamics. The Holocene was marked by rapid climate changes—from moist (African humid period), during which forests and woodlands expanded [69-71], towards increasingly drier conditions around 4000–6000 YBP, concomitantly with the buffalo population decline [31]. This considerable and quite rapid climatic shift likely occurred on a large spatial scale, as declines in the African buffalo effective population size were recorded in several African regions (East and South Africa) [31,72]. Climate change in the Holocene was likely severe enough to have considerably impacted the African buffalo [73], leading to population fragmentation.

In addition to the climate hypothesis, human populations likely had an impact on the buffalo population fragmentation process. Indeed, according to Heller *et al.* [31], during the human Neolithic revolution, sub-Saharan human populations started to increase while, inversely, the African Cape buffalo population started to significantly decrease. This human impact would have been enhanced around 2000 years ago [74]. Around that time, the first southern African states were established by prosperous cattle herding people who adopted crop farming. Cattle husbandry led to the development of complex societal and political systems [74-78]. By 1500 A.D., most of southern Africa was governed by societies managing large domestic livestock herds (cattle, goat and sheep). Cattle populations increased rapidly following the Neolithic revolution [72]. In this setting, it is likely that the southern African buffalo population progressively suffered from competition with livestock for food resources and that significant discontinuities appeared in its initial distribution range. Moreover, it is also possible that aridification events of the Holocene drove humans and wildlife into closer contact around water resources, thus increasing ecological competition. Direct buffalo hunting, as a food supply and/or to reduce competition with domestic livestock species, was likely another important factor.

The divergence between the N and C buffalo clusters could be explained by this break of continuity in the landscape matrix due to both aridification and progressive human/cattle population growth, and/or by potential overhunting. At the regional level, the combined effects of rapidly expanding human activities and sudden climatic changes may have been primary forces that fragmented a previously panmictic population and shaped the current genetic structure of African buffalo. In addition, N and C clusters are hypothetised to share a common ancestral population. The Y-chromosomal minimum spanning reconstruction identified a haplogroup, namely {5, 5, 7}, that was present in the three clusters and occurred in a central position, while displaying the highest frequencies of appearance (0.123). Its presence in all clusters could support our assumption of a common ancestral panmictic buffalo population that recently experienced fragmentation. The isolation process persisted and amplified during the 20^th^ century due to human and cattle population growth. A good example is central Zimbabwe, a plateau that offers excellent grazing between the Zambezi and Limpopo rivers. In this area, wild herbivores, including buffalo, are generally considered as disease reservoirs and were thus controlled or even eradicated over the last century to protect cattle on commercial farms.

A third potential explanatory factor of the isolation of N and C clusters concerns the spatial arrangement of the water system. Indeed, the African buffalo is a highly water dependent species and the large-scale distribution and regime of rivers may well explain the observed genetic structure. This seems possible since the ranges of N and C clusters roughly span the Zambezi and the Limpopo river basins, as well as Rovuma, Pungoe, Save and other river basins. Colonisation of southern Africa by buffalo from an eastern core (Uganda [17]) may have followed the primary river networks, thus leading to the emergence of two different genetic clusters. This hypothesis is not unlikely, but our recent study based on mtDNA [17] dated the buffalo colonisation of southern Africa at around 44,000 and 66,000 years ago. This precedes the microsatellite-estimated splitting time between the N and C clusters by several tens of thousands of years. The hypothesis of a population fragmentation associated with the rapid human demographic expansion and climatic changes a few thousands years ago therefore seems much more likely.

### Impact of translocations on the genetic structure of N and C clusters

Within the three identified clusters, discrepancies between the genetic affiliation of some individuals and their geographical origins were observed (Figures 4 and 5). Natural migration and/or translocation of individuals could both be responsible for buffalo genetic patterns observed in southern Africa. As already mentioned, African buffalo is known to have a good dispersal capacity, as demonstrated in previous studies [13,15,24-26]. Nevertheless, the immigration rates per generation between our three clusters appeared to be low, reaching a maximum of two individuals per generation. Moreover, since a disease-free zone for commercial cattle farming was set up in central Zimbabwe, natural buffalo migration between northern and southern Zimbabwe is very limited. Buffalo now still seem to migrate in smaller numbers across areas with fairly substantial human settlements, e.g. along rivers, but the reduced connectivity between the protected areas seems to affect its dispersal.

**Figure 7:**
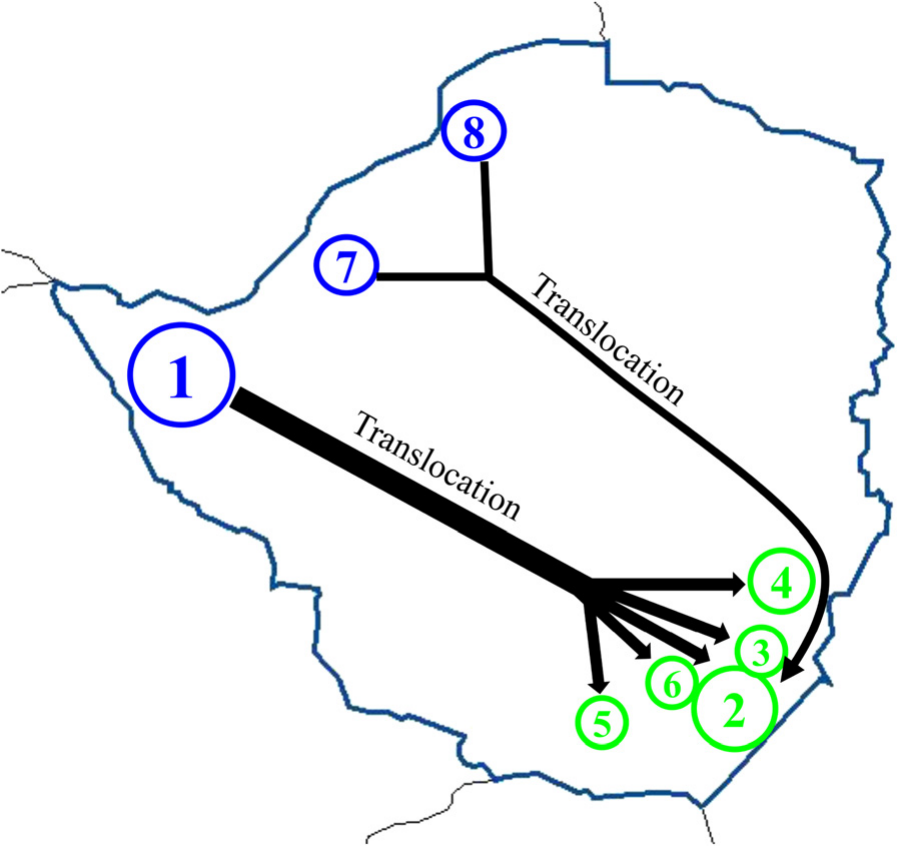
**Representation of known translocation events between Northern and Central protected areas of Zimbabwe.** 1. Hwange, 2. Gonarezhou, 3. Malilangwe, 4. Save Valley, 5. Bubye Valley, 6. Nuanetsi, 7. Chizarira, 8. Charara. Blue: Northern cluster, Green: Central cluster.

Translocations over the last century seem to best explain the discrepancies observed in the identified genetic pattern highlighted in this study. In fact, records indicate that buffaloes from Malilangwe (C cluster) were primarly, but not exclusively, stocked with buffaloes from Hwange (N cluster) (C. Foggin, pers. comm.). Moreover, individuals from three different localities of the N cluster (Hwange, Chizarira and Charara) were moved to Gonarezhou (C cluster) (C. Foggin, pers. comm.). Save Valley, Bubye Valley and Nuanetsi buffalo populations, all located near Gonarezhou and Malilangwe (C cluster), were restocked from Hwange (N cluster—C. Foggin, pers. comm.) (Figure 7). Consequently, translocations seem to be the most plausible explanation for the lower *D*_*EST*_ estimates obtained between Malilangwe (C cluster) and Hwange/Victoria Falls/Chobe/Mana Pools complex (N cluster). In addition, translocation would also explain the very low *D*_*EST*_ values between Hwange (N cluster) and all C sampled localities, except for Gorongosa and Limpopo. Buffalo from Hwange, Lusulu (south of Chizarira) and Matusadona were selected to form herds free of foot-and-mouth disease, which is transmissible to cattle. These buffalo were then bred and subsequently transferred to many regions of Zimbabwe, including commercial wildlife properties adjacent to PAs (protected areas) within this country (C. Foggin, pers. comm.) [79]. The present study revealed a marked impact of translocations on the regional genetic structure of the studied species. At a larger scale, the present findings highlighted the issue and impacts of translocations regarding species conservation management. For endangered species, translocation is often considered essential to restore genetic diversity of highly isolated populations threatened with extinction and in areas where the species is extinct [6]. Nevertheless, for least vulnerable species such as the African buffalo (Least Concern- IUCN v.2013.2, downloaded on 8 December 2013), greater consideration should be given to cluster affiliations when planning translocations, if the relevant information is available. Failure to do so could interfere with local adaptations to specific environmental conditions. Not taking in consideration contemporary micro-evolutionary change, often associated to human activities (ex. habitat fragmentation), may lead to ineffective or even detrimental management practices [80]. This consideration relates to the choice of the most appropriate options for improving the conservation management of species populations regarding their environmental, behavioural and genetic specificities, as well as their conservation status (http://www.iucnredlist.org/). The ecological and evolutionary consequences of resource management decisions are further discussed in the review of Ashley *et al.* [80], advocating evolutionary enlightened management.

### The unique history of Hluhluwe-iMfolozi buffaloes (Southern cluster)

Hluhluwe-iMfolozi is a unique protected area as it has been completely isolated for about 100 years. Only 75 individuals were reported in this region in 1929, and this population may have been reduced partly as a result of the Nagana campaigns against the disease carrying tsetse fly (1919–1950) [20]. The current population (*N* = ± 4000 [21]) directly stems from these survivors, which could explain the recent bottleneck signal observed. Our genetic results support this isolation and population decrease event. Indeed, the pairwise *F*_*ST*_ and *D*_*EST*_ indices between the Southern cluster and the two other clusters had high values (more than twofold higher than between the N and C clusters), suggesting substantial isolation of the Southern cluster.

Nevertheless, and surprisingly, the Hluhluwe-iMfolozi buffalo population still harbours high estimated allelic richness, heterozygosity and genetic diversity. Note, however, that all of those estimates were lower as compared to those of the two other clusters, with *F*_*IS*_ values two- and fourfold higher than for the C and the N clusters, respectively. The gene flow disruption due to a recent isolation (±17 generations) was likely responsible for the observed genetic erosion, with signs of inbreeding depression (*F*_*IS*_). In this setting, future increases in genetic drift within the S population will probably lead to a more pronounced loss of genetic diversity as compared to the current situation, although faintly detectable by now. O’Ryan [20] similarly concluded that the Kruger population has retained most of its original variants present 100 years ago, in contrast to the Hluhluwe-iMfolozi, Addo and St Lucia populations which lost their variants through genetic drift. This trend is particularly clear when considering the number of private alleles. Unique allele variants were lowest in the S cluster (*P*_*a*_ = 2), while eight were noted in the C cluster. The high genetic variation that is still being recorded may be explained by a historically large population size, with current buffalo population sizes still above the critical threshold (as discussed hereafter).

### Within-cluster genetic diversity, differentiation and bottleneck signals

The N cluster had the highest mean number of alleles, private alleles (*P*_*a*_ = 13), heterozygosity and genetic diversity estimates, while displaying the lowest inbreeding coefficient. This may have been the result of a wider geographical distribution and higher population size estimates compared to the two other clusters (see next section). Moreover, in contrast with the two other clusters, the N cluster did not display any recent bottleneck signal. The C cluster (which includes the Kruger and surrounding protected areas) is characterised by a significant signal of recent bottleneck. Buffaloes in the Kruger area were highly affected by rinderpest during the last decade of the 19^th^ century, with a high mortality rate and a small number of survivors reported in 1902 [81]. The recent bottleneck signal may thus be associated with this rinderpest outbreak. However, apart from the study of Heller *et al.* [31] which highlighted a bottleneck signal caused by the rinderpest epidemic in the late 19^th^ century, all other studies conducted on that topic demonstrated that the outbreak did not seem to impact the genetic variability of this population [14,20,82,83]. As previously proposed by van Hooft *et al.* [15], the absence of genetic erosion also observed within our study could be explained by high gene flow between the C sampled localities, thus re-establishing the lost genetic variability. This assumption is supported by observed low *F*_*ST*_ and *D*_*EST*_ values for the pairwise sampling localities of the C cluster (Table 5), indicating high dispersal events between them. The high genetic variability in the C cluster is commonly recognised as being the result of two different features: (i) a very high ancestral population size [13,20], and (ii) a capacity to maintain a non-critical population size through a relatively high rate of increase [21] and a good dispersal ability [20,22,26,30,84]. This is not unlikely as the buffalo is vagile, with bachelor bulls readily travelling large distances between herds, with entire herds sometimes moving and settling away from their initial ranges [25]. Groups of young females (less than 3 years-old) are often reported to escape through the Kruger fence into communal areas (R. Bengis, pers. comm.). This dispersive behavior may have helped the Southern African Cape buffalo population to recover its population size within 20–30 years after the rinderpest outbreaks [13,15,29,84]. Recolonization of the initial range from neighbouring protected areas, as also proposed by van Hooft *et al.* [15] regarding the Kruger PA, is the most plausible explanation for the high genetic diversity observed within the different sampling localities of the C cluster [15,85].

Within each of the N and C clusters, the SL showed a low level of genetic differentiation and high heterozygosity, comparable to the values obtained in previous studies on the African buffalo. This extent of genetic diversity in African buffalo is particularly high as compared to estimates in other large African savanna ungulates [13,15,22]. At the SL scale, the *D*_*EST*_ and traditional pairwise *F*_*ST*_ statistical findings both lead to the same conclusion. Almost all *D*_*EST*_ between sampling sites were extremely low within the C and N clusters (mean 0.007 and 0.022, respectively). This suggests the possibility of high gene flow within each of these clusters, as proposed hereafter. Nevertheless, in the N cluster, the mean *D*_*EST*_ were sixfold higher than in the C cluster, which may be partly explained by the higher *D*_*EST*_ between Niassa and all the other sampling sites (mean 0.050). This may be attributed to the geographical distance separating Niassa from all other sampling localities, as indicated by the significant IBD.

Interestingly, another complementary explanation may be linked to the very high *D*_*EST*_ values of the Marromeu complex, i.e. a network of protected areas (National Reserve and Hunting Areas) included within the N cluster, as compared to the other SLs of the N cluster (mean *D*_*EST*_ = 0.076) but also to SLs of the two other clusters (mean *D*_*EST*_ = 0.100). The Marromeu complex hosts a high number of buffaloes (>10,300 individuals [35]) that have been relatively isolated for several centuries within a particular biotope, i.e. swamps in the Zambezi delta region (C. Lopes Pereira, pers. comm.). When translocated, their adaptation to the typical habitats of surrounding buffalo populations (Miombo ecosystem) is very slow and lengthy (C. Lopes Pereira, pers. comm.) [80]. These animals thus appear to be adapted to floodplains. Moreover, in 1996, the Marromeu buffalo population size was estimated to be about 2500 individuals [35], indicating that this population increased fourfold between then to now. While significant genetic drift could be detected within the Hluhluwe-iMfolozi PA, which experienced a strong founder event, the Marromeu buffalo population decline and re-growth does not seem to have led to a substantial loss of heterozygosity and differentiation (Figure 6B). However, due to the relative isolation of this specific sampling area, and the already high observed-*D*_*EST*_ values, genetic drift may occur in the future.

### Long-term species and habitat conservation

The genetically effective population size (*N*_*e*_) estimates are generally much smaller than the population census size (*N*_*c*_) [86,87]. Considering the African buffalo, it is recognised that *N*_*e*_ ranges between 10 and 30% of the *N*_*c*_ [20,9]. The *N*_*e*_ of the N cluster was estimated to range between 7000 and 25,000, those of the S cluster between 600 and 2000, and those of the C cluster between 3000 and 10,000. The corresponding expected *N*_*c*_ is therefore estimated to range from a few tens of thousands to several hundreds of thousands according to the studied regions (*N*_*c*__Northern_: 23,000 to 250,000, *N*_*c*__Southern_: 2000 to 20,000, *N*_*c*__Central_: 10,000 to 100,000, respectively). Aerial counts, although potentially subject to major bias [88,89], were shown to provide the most reliable estimates of buffalo savanna populations. Based on those studies [21,32,34-38,90], a current estimation of the global extant population size of each of the three clusters could be evaluated at about 90,000, 4000 and 50,000 individuals for the N, S and C clusters, respectively (Table 1). All buffalo numbers estimated by aerial counts were within the current *N*_*c*_ estimated ranges of each of the clusters. This suggests that the southern African buffalo populations are relatively healthly, as also supported by the high level of genetic diversity observed within each of the genetic clusters studied. Moreover, the total estimated population census sizes (*N*_*c*_) reported hereafter represent underestimations of the real current population sizes because all protected areas in southern Africa were not sampled in this study, and also because buffaloes roam outside of protected areas (75% in PAs versus 25% outside PAs [10]). Even though they often occur at low density and population size, buffaloes roaming outside of PAs are commonly considered to play a crucial role in linking buffalo populations between PAs.

In order to maintain historical levels of genetic variability at microsatellite loci for the purpose of long-term conservation, movements between confined protected areas should be facilitated. Hence, the recent development of transfrontier conservation areas (TFCAs) in southern Africa aims at establishing vast ecosystems encompassing protected areas of various statuses (e.g. national parks, conservancies) and communal land so as to fulfil development and conservation objectives. These entities are designed to promote ecological connectivity between protected areas (e.g. plan to create the Sengwe Corridor between Kruger and Gonarezhou PAs in the Great Limpopo TFCA) to ease wildlife dispersal and achieve critical population sizes [91]. Regular translocations between areas within clusters could also be supported in order to maintain the genetic diversity [15,20], particularly for entirely or partially fenced protected areas (e.g. Malilangwe, Hluhluwe-iMfolozi, etc.). Moreover, to respect the natural behaviour of the African buffalo, it has been suggested that preference be given to the translocation of males since they have the highest migration rate per generation (males: 100% per generation [9], females: 15% estimated during a 20-month tracking period [26], 2% estimated monthly [92]). In the absence of such measures, effective population sizes are exposed to decrease through genetic erosion. In the near future, the current high genetic diversity observed in this study could deteriorate due to population fragmentation and reduction in the absence of proper conservation management [80].

## Conclusion

The present study supports assumptions that both ancient (over few thousand years ago) and recent (over 100 years ago) population fragmentations had an impact on the genetic structure of the African buffalo, leading to the identification of three distinct clusters in southern Africa. Our results highlight low levels of genetic differentiation within and between each cluster/sampling locality, each with high genetic diversity and low inbreeding coefficient estimates. Even though differentiation was low, migration between clusters was shown to be relatively limited. Connectivity between protected areas is therefore necessary to ensure the conservation of African mammal diversity, as supported by the transfrontier conservation area (TFCA) initiative. Moreover, prior to any translocation operation, it is essential to consider the environmental, behavioural, genetic and conservation specificities in order to mitigate further genetic erosion.

## Availability of supporting data

The data set supporting the results of this article is available in the Dryad repository, doi:10.5061/dryad.8f409 (http://datadryad.org/).

## Additional files

Additional file 1: Table S1. Summary table of the 14 autosomal and 3 Y-chromosomal microsatellite loci used in this study. The table reports the position of the loci on the cattle chromosomes, allelic range, number of alleles and private alleles for each studied locus. Additional file 2: Figure S1. Representation of competing scenarios designed and tested using approximate Bayesian computation (ABC) analysis. This analysis was based on a matrix including individuals displaying a probability of belonging to one of the two clusters over 0.9 (STRUCTURE software). Additional file 3: Table S2. Prior distribution of parameters used in our ABC analysis. Additional file 4: Figure S2. Assessment of the goodness-of-fit of the model parameter posterior combination (scenario 3- Figure 2). Datasets simulated with the prior distributions of parameters and the observed data, as well as datasets from the posterior predictive distribution, are represented on each plane of the principal component analysis (PCA). Additional file 5: Figure S3. Results of the Bayesian clustering analysis with STRUCTURE software. The first figure reports the Δ*K* values calculated according to Evanno *et al.* [55], while the second figure reports the tested K in function of the mean ln (PD). Additional file 6: Table S3. Different genetic parameters estimated at each cluster at each locus (GENECLASS2 software). Additional file 7:
**Complementary IM analysis details.**
Additional file 8: Figure S4. Linear regressions between patch areas of each SL (expressed as log km^2^) and allelic richness *A*_*R*_, mean expected heterozygosity *H*_*E*_, mean pairwise *D*_*EST*_ and mean pairwise *F*_*ST*_. *R* ^*2*^: coefficients of determination.
