# Erratum to: genetic structure of fragmented southern populations of African Cape buffalo (Syncerus caffer caffer)

**DOI:** 10.1186/s12862-016-0585-4

**Published:** 2016-01-20

**Authors:** Nathalie Smitz, Daniel Cornélis, Philippe Chardonnet, Alexandre Caron, Michel de Garine-Wichatitsky, Ferran Jori, Alice Mouton, Alice Latinne, Lise-Marie Pigneur, Mario Melletti, Kimberly L. Kanapeckas, Jonathan Marescaux, Carlos Lopes Pereira, Johan Michaux

**Affiliations:** Departement of Life Sciences-Conservation Genetics, University of Liège, Liège, Belgium; Centre de Coopération Internationale en Recherche Agronomique pour le Développement (CIRAD), Montpellier, France; Fondation Internationale pour la Gestion de la Faune (IGF), Paris, France; Centre de Coopération Internationale en Recherche Agronomique pour le Développement (CIRAD)-RP-PCP, University of Zimbabwe, Harare, Zimbabwe; Mammal Research Institute, Department of Zoology and Entomology, University of Pretoria, Pretoria, South Africa; Department of Biological Sciences, University of Zimbabwe, Harare, Zimbabwe; Department of Animal Science and Production, Botswana College of Agriculture, Gaborone, Botswana; Institut des Sciences de l’Evolution-CNRS-IRD, Université de Montpellier 2, Montpellier, France; Department of Parasitology, Faculty of Veterinary Medicine, Kasetsart University, Bangkok, Thailand; Research Unit in Environmental and Evolutionary Biology, University of Namur, Namur, Belgium; Independent researcher, Via Di Villa Chigi, Rome, Italy; Department of Genetics and Biochemistry, Clemson University, Clemson, USA; Wildlife Conservation Society, New York, USA

## Erratum

The original version [1] of this article unfortunately contained a mistake in the acknowledgements section in that the funding information was not included in the HTML and PDF versions of this article. The corrected acknowledgements section with the funding information present is provided below:
