# Supplementary material for: Genetic structure of fragmented southern populations of African Cape buffalo (Syncerus caffer caffer)
Source: BMC Evol Biol. 2014 Nov 1;14:203. doi: 10.1186/s12862-014-0203-2 (PMC4232705; doi:10.1186/s12862-014-0203-2)
Supplement: Additional file 1: Table S1. — Summary table of the 14 autosomal and 3 Y-chromosomal microsatellite loci used in this study. The table reports the position of the loci on the cattle chromosomes, allelic range, number of alleles and private alleles for each studied locus. [file 12862_2014_203_MOESM1_ESM.docx]

| Locus | Primer sequences (5’-3’) | | Cattle Chr number | Allelic Range | Number of alleles | Number of private alleles | References |
| --- | --- | --- | --- | --- | --- | --- | --- |
| *TGLA227-FAM* | F | CgA ATT CCA AAT CTg TTA ATT TgC T | 18 | 70-76 | 4 | 0 | [32] |
|  | R | ACA gAC AgA AAC TCA ATg AAA gCA |  |  |  |  |  |
| *TGLA263-PET* | F | CGAATTCCAAATCTGTTAATTTGCT | 3 | 112-130 | 9 | 2 | [32] |
|  | R | ACAGACAGAAACTCAATGAAAGCA |  |  |  |  |  |
| *ETH225-VIC* | F | gAT CAC CTT gCC ACT ATT TCC T | 5 | 133-137 | 2 | 0 | [33] |
|  | R | ACA TgA CAg CCA gCT gCT ACT |  |  |  |  |  |
| *ABS010-NED* | F | GGAGTGAGACAGGGGTGTGT | 13 | 168-194 | 10 | 3 | [32] |
|  | R | TGGCATCCATTCATCCATCT |  |  |  |  |  |
| *BM1824-FAM* | F | gAg CAA ggT gTT TTT CCA ATC | 1 | 169-199 | 14 | 1 | [33] |
|  | R | CAT TCT CCA ACT gCT TCC TTg |  |  |  |  |  |
| *ETH010-NED* | F | gTT CAg gAC Tgg CCC TgC TAA CA | 5 | 204-208 | 3 | 0 | [33] |
|  | R | CCT CCA gCC CAC TTT CTC TTC TC |  |  |  |  |  |
| *SPS115-PET* | F | AAAGTGACACAACAGCTTCTCCAG | 15 | 223-255 | 15 | 2 | [33] |
|  | R | AACGAGTGTCCTAGTTTGGCTGTG |  |  |  |  |  |
| *INRA006-NED* | F | Agg AAT ATC TgT ATC AAC CTC AgT C | 3 | 107-127 | 8 | 0 | [32] |
|  | R | CTg AgC Tgg ggT ggg AgC TAT AAA TA |  |  |  |  |  |
| *BM4028-FAM* | F | ACg gAA gCA gCA TCT CTT AC | 29 | 120-134 | 4 | 1 | [32] |
|  | R | ATg gAA ACA Tgg TCT CCT gC |  |  |  |  |  |
| *INRA128-NED* | F | TAA gCA CCg CAC AgC AgA TgC | 1 | 166-182 | 9 | 3 | [32] |
|  | R | AgA CTA gTC Agg CTT CCT AC |  |  |  |  |  |
| *CSSM19-VIC* | F | TTg TCA gCA ACT TCT TgT ATC TTT | 1 | 128-158 | 13 | 1 | [32] |
|  | R | TgT TTT AAg CCA CCC AAT TAT TTg |  |  |  |  |  |
| *AGLA293-PET* | F | GAAACTCAACCCAAGACAACTCAAG | 5 | 200-258 | 23 | 5 | [32] |
|  | R | ATGACTTTATTCTCCACCTAGCAGA |  |  |  |  |  |
| *ILSTS026-FAM* | F | CTg AAT Tgg CTC CAA Agg CC | 2 | 143-167 | 12 | 3 | [32] |
|  | R | AAA CAg AAg TCC Agg gCT gC |  |  |  |  |  |
| *DIK020-VIC* | F | AAC CAg TAA TCg TgA gAg gA | 10 | 162-208 | 18 | 2 | [32] |
|  | R | AAg AAA gTC CCT ACC ATg Ag |  |  |  |  |  |
| *UMN1113-NED* | F | ACAGCACTTCTTAACAAAGC | Y | 131-159 | See Table 2 | NA | [34] |
|  | R | TAGCCACACATCATGTTC |  |  |  |  |  |
| *INRA189-PET* | F | TACACGCATGTCCTTGTTTCGG | Y | 138-166 | See Table 2 | NA | [34] |
|  | R | CTCTGCATCTGTCCTGGACTGG |  |  |  |  |  |
| *UMN0304-FAM* | F | TGATATTCACAAGGCCGCTG | Y | 205-227 | See Table 2 | NA | [34] |
|  | R | GGCTGTGGTATACTATGGAG |  |  |  |  |  |

Additional file- Table 1. Summary table of the 14 autosomal and 3 Y-chromosomal microsatellite loci used in this study. The table reports the position of the loci on the cattle chromosomes, allelic range, number of alleles and private alleles for each studied locus.
