# Supplementary material for: Genetic structure of fragmented southern populations of African Cape buffalo (Syncerus caffer caffer)
Source: BMC Evol Biol. 2014 Nov 1;14:203. doi: 10.1186/s12862-014-0203-2 (PMC4232705; doi:10.1186/s12862-014-0203-2)
Supplement: Additional file 2: Figure S1. — Representation of competing scenarios designed and tested using approximate Bayesian computation (ABC) analysis. This analysis was based on a matrix including individuals displaying a probability of belonging to one of the two clusters over 0.9 (STRUCTURE software). [file 12862_2014_203_MOESM2_ESM.docx]

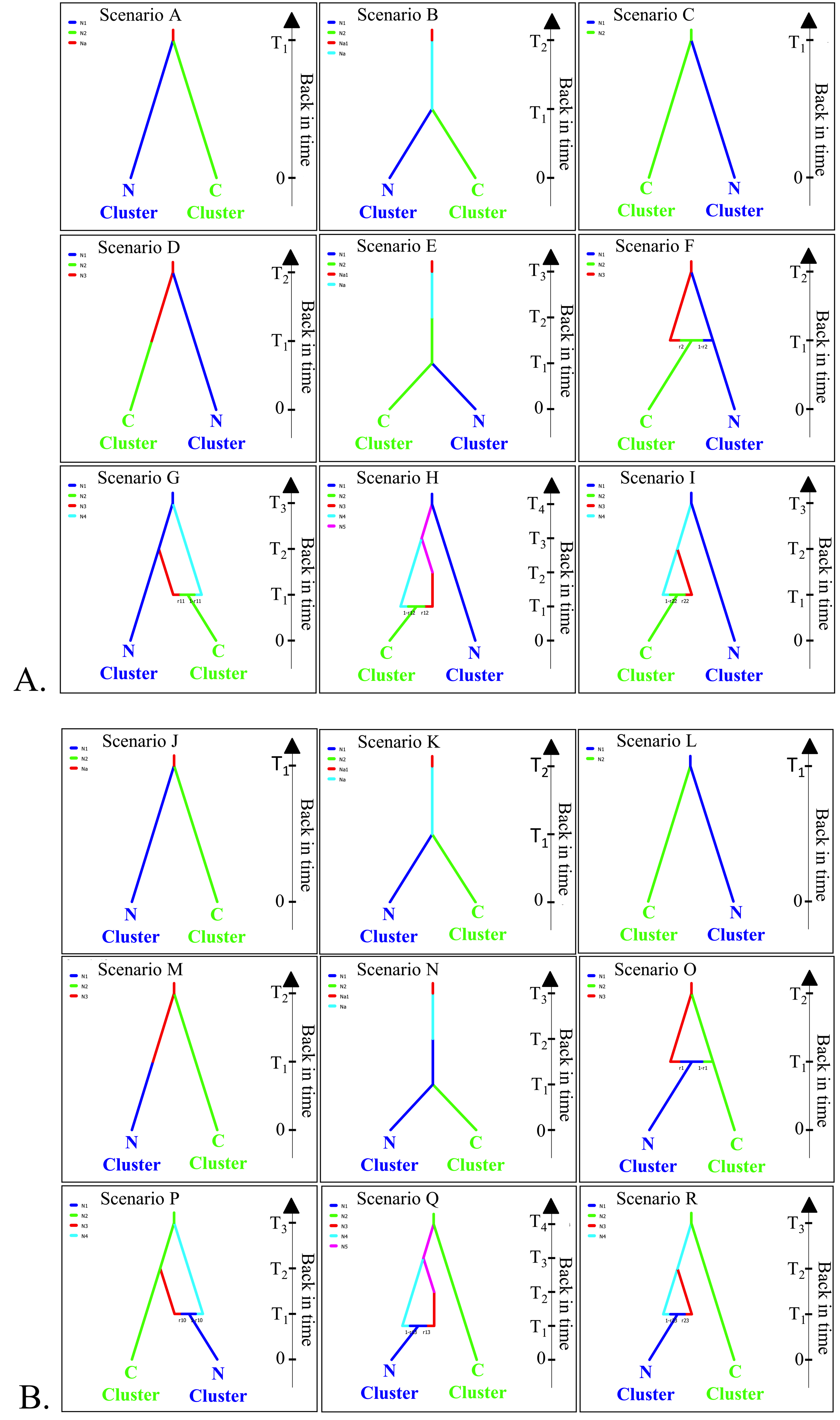


A. competing scenarios designed to test the origin of the Central cluster, B. competing scenarios designed to test the origin of the Northern cluster. N_i_ corresponds to the effective population size of each cluster, and T_i_ corresponds to the time since divergence. The following conditions were considered: T_1_<T_2_, T_2_<T_3_ and T_3_<T_4_, with 0 being the sampling date. Abbreviations are as follows: C; Central cluster, N; Northern cluster, AP_i_; Ancestral populations, ra; admixture rate. We built scenarios that made biological sense, while first testing a series of basic scenarios with simple binary splits (A to E and J to N). They were supplemented with a series of scenarios including admixture events (F to I and O to R). The purpose was to test the origin of each of the two clusters. They focused on whether the current clusters originated from the differentiation of an ancestral panmictic population or if southern Africa experienced multiple buffalo population differentiation events, leading to (through admixture or not) the currently existing two clusters. In detail, we tested: i) an origin for both clusters from a hypothetical ancestral population without admixture event (scenarios A, B, D and J, K, M); ii) an origin of one cluster resulting from the differentiation of an ancestral subset of one of our identified cluster without admixture event (scenarios C, E and L, N); iii) an origin from hypothetical ancestral populations incorporating admixture events. In this latter case, we tested the origin of our clusters from the admixture: a) of a hypothetical ancestral population with the ancestors of one of our identified clusters (scenarios F and O); b) of distinct differentiated populations originating from the ancestor of one of our identified clusters (scenarios G, H, I and P, Q, R).
