# Supplementary material for: Genetic structure of fragmented southern populations of African Cape buffalo (Syncerus caffer caffer)
Source: BMC Evol Biol. 2014 Nov 1;14:203. doi: 10.1186/s12862-014-0203-2 (PMC4232705; doi:10.1186/s12862-014-0203-2)
Supplement: Additional file 3: Table S2. — Prior distribution of parameters used in our ABC analysis. [file 12862_2014_203_MOESM3_ESM.docx]

| **Parameter** | **Distribution** | **Min.** | **Max.** | **Step** |
| --- | --- | --- | --- | --- |
| **Effective population size** |  |  |  |  |
| N_1_, N_2_, N_3_, N_4_, N_5_, Na, Na_1_ | Uniform | 10 | 15,000 | 1 |
| **Time of events (in generations backward in time)** | Log-uniform |  |  |  |
| T_i_ (Conditions: T_1_<T_2_, T_2_<T_3_, T_3_<T_4_) |  | 10 | 20,000 | 1 |
| **Admixture rate (ra)** | Uniform | 0.001 | 0.999 | 0.001 |
| **Mutation model parameters** |  |  |  |  |
| Mean mutation rate | Uniform | 0.0001 | 0.001 |  |
| Mean coefficient P | Uniform | 0.1 | 0.3 |  |
| Mean SNI rate | Log-uniform | 1.00 ^-008^ | 1.00 ^-005^ |  |

- Additional file- Table 2. Prior distribution of parameters used in our ABC analysis
