# Supplementary figures and images for: Genetic structure of fragmented southern populations of African Cape buffalo (Syncerus caffer caffer)
Source: BMC Evol Biol. 2014 Nov 1;14:203. doi: 10.1186/s12862-014-0203-2 (PMC4232705; doi:10.1186/s12862-014-0203-2)

Components 1 and 2

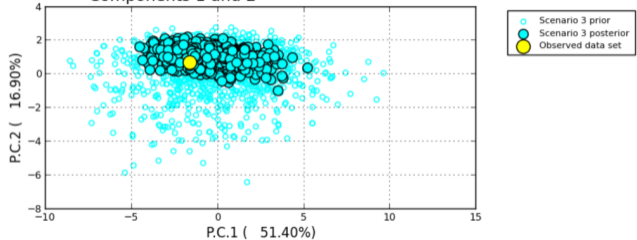

Supplement: Additional file 4: Figure S2. — Assessment of the goodness-of-fit of the model parameter posterior combination (scenario 3- Figure 2). Datasets simulated with the prior distributions of parameters and the observed data, as well as datasets from the posterior predictive distribution, are represented on each plane of the principal component analysis (PCA). [file 12862_2014_203_MOESM4_ESM.pdf]

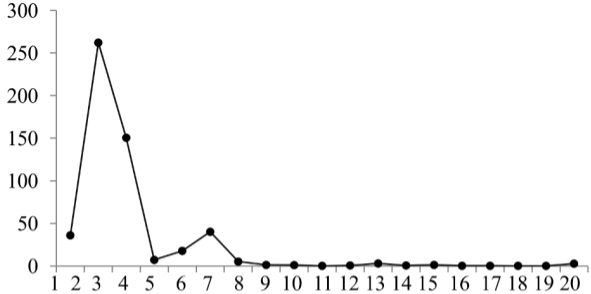

1 2 3 4 5 6 7 8 9 10 11 12 13 14 15 16 17 18 19 20

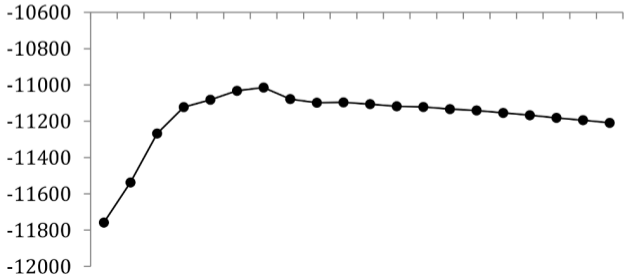

Supplement: Additional file 5: Figure S3. — Results of the Bayesian clustering analysis with STRUCTURE software. The first figure reports the ΔK values calculated according to Evanno et al. [55], while the second figure reports the tested K in function of the mean ln (PD). [file 12862_2014_203_MOESM5_ESM.pdf]

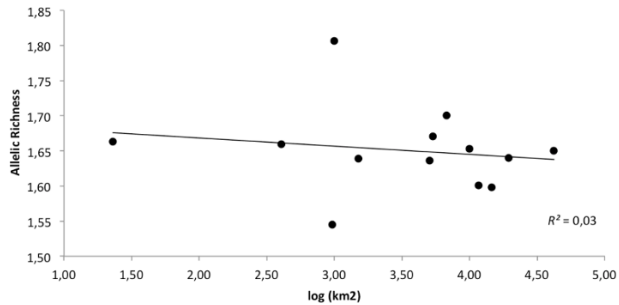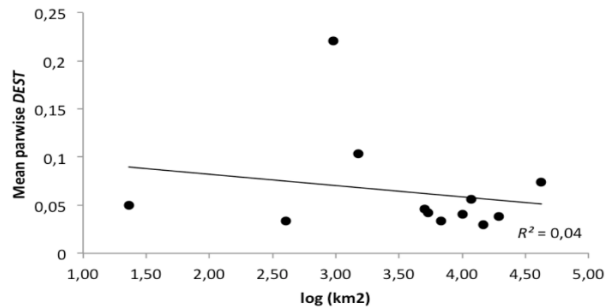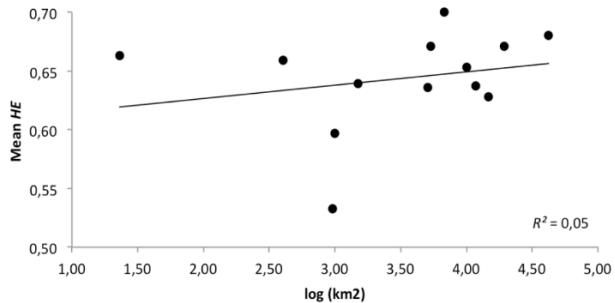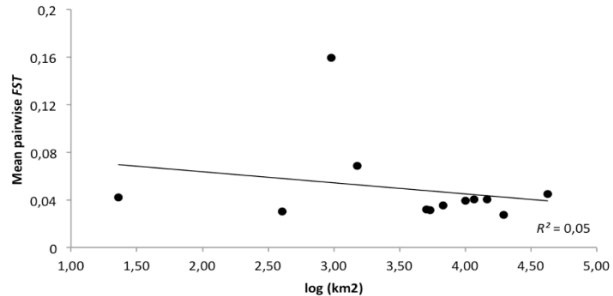

Supplement: Additional file 8: Figure S4. — Linear regressions between patch areas of each SL (expressed as log km2) and allelic richness A R, mean expected heterozygosity H E, mean pairwise D EST and mean pairwise F ST. R 2: coefficients of determination. [file 12862_2014_203_MOESM8_ESM.pdf]
