# Supplementary material for: Genetic structure of fragmented southern populations of African Cape buffalo (Syncerus caffer caffer)
Source: BMC Evol Biol. 2014 Nov 1;14:203. doi: 10.1186/s12862-014-0203-2 (PMC4232705; doi:10.1186/s12862-014-0203-2)
Supplement: Additional file 6: Table S3. — Different genetic parameters estimated at each cluster at each locus (GENECLASS2 software). [file 12862_2014_203_MOESM6_ESM.docx]

|  | Number of alleles | | | Heterozygote proportion | | | Gene diversity (Nei) | | |
| --- | --- | --- | --- | --- | --- | --- | --- | --- | --- |
|  | Northern | Southern | Central | Northern | Southern | Central | Northern | Southern | Central |
| *TGLA227* | 4 | 3 | 4 | 0.324 | 0.516 | 0.486 | 0.303 | 0.597 | 0.511 |
| *TGLA263* | 8 | 5 | 7 | 0.741 | 0.839 | 0.661 | 0.750 | 0.669 | 0.633 |
| *ETH225* | 2 | 2 | 2 | 0.546 | 0.161 | 0.440 | 0.471 | 0.151 | 0.489 |
| *ABS010* | 9 | 7 | 7 | 0.667 | 0.774 | 0.688 | 0.640 | 0.766 | 0.651 |
| *BM1824* | 13 | 6 | 14 | 0.786 | 0.586 | 0.742 | 0.876 | 0.739 | 0.818 |
| *ETH010* | 3 | 2 | 3 | 0.224 | 0.355 | 0.282 | 0.201 | 0.373 | 0.280 |
| *SPS115* | 15 | 13 | 13 | 0.905 | 0.839 | 0.806 | 0.887 | 0.823 | 0.839 |
| *INRA006* | 8 | 5 | 8 | 0.785 | 0.548 | 0.632 | 0.809 | 0.700 | 0.653 |
| *BM4028* | 4 | 2 | 3 | 0.213 | 0.355 | 0.200 | 0.220 | 0.337 | 0.213 |
| *INRA128* | 8 | 6 | 7 | 0.598 | 0.333 | 0.592 | 0.652 | 0.381 | 0.596 |
| *CSSM19* | 13 | 8 | 11 | 0.815 | 0.742 | 0.832 | 0.854 | 0.715 | 0.833 |
| *AGLA293* | 20 | 12 | 20 | 0.898 | 0.710 | 0.846 | 0.922 | 0.825 | 0.912 |
| *ILSTS026* | 10 | 9 | 11 | 0.852 | 0.484 | 0.824 | 0.857 | 0.564 | 0.855 |
| *DIK020* | 16 | 8 | 18 | 0.828 | 0.536 | 0.864 | 0.910 | 0.639 | 0.914 |
| Mean | 9.500 (5.360) | 6.286 (3.518) | 9.143 (5.614) | 0.668 (0.264) | 0.591 (0.205) | 0.657 (0.224) |  |  |  |

Additional file- Table 3. Different genetic parameters estimated at each cluster at each locus (GENECLASS2 software).
