# Supplementary material for: Genetic structure of fragmented southern populations of African Cape buffalo (Syncerus caffer caffer)
Source: BMC Evol Biol. 2014 Nov 1;14:203. doi: 10.1186/s12862-014-0203-2 (PMC4232705; doi:10.1186/s12862-014-0203-2)
Supplement: Additional file 7: — Complementary IM analysis details. [file 12862_2014_203_MOESM7_ESM.docx]

Complementary IM analysis details:

**Method:** IM software (Hey & Nielsen, 2004; Hey, 2005), applying the Isolation with Migration model, was used to assess different molecular demographic parameters from the two microsatellite matrices between the two studied populations (i.e. rates of gene flow in both directions, ancestral and contemporary effective population sizes and population-split time), scaled by the mutation rate. As IM does not assume that gene flow and genetic drift are at equilibrium, it is highly suitable for assessing recently diverged populations that share alleles due to both gene flow and ancestral polymorphism. Limits were adjusted to obtain better distribution ranges. Prior distributions were chosen in order to include all or most ranges over which the posterior density was non-trivial, while applying the Stepwise Mutation Model (SSM) for each microsatellite locus. A burn-in MCMC chain of indefinite length was initiated and run for a sufficient length of time that there would be no obvious trends in the trend line plots. The genealogy at the end of this burn-in run was used as a starting point for the sampling MCMC run. A hundred thousand independent genealogies were sampled by running multiple chains (10) with a short burn-in period that was long enough to obtain independent starting points.

**Result:** Convergence was not achieved when the IM software was run on both microsatellite matrices, probably because the IM model could not account for the low genetic differentiation among the studied clusters and the high genetic diversities within clusters. This was supported by the AMOVA results, showing a very high variation percentage within clusters (87.40%) as compared to the variation among them (12.60%).

Hey, J. (2005). On the number of New World founders: a population genetic portrait of the peopling of the Americas. *PLoS biology*, *3*(6), e193. doi:10.1371/journal.pbio.0030193

Hey, J., & Nielsen, R. (2004). Multilocus methods for estimating population sizes, migration rates and divergence time, with applications to the divergence of Drosophila pseudoobscura and D. persimilis. *Genetics*, *167*(2), 747–60. doi:10.1534/genetics.103.024182
